# Supplementary material for: Selection and Development of Nontoxic Nonproteolytic Clostridium botulinum Surrogate Strains for Food Challenge Testing
Source: Foods. 2022 May 27;11(11):1577. doi: 10.3390/foods11111577 (PMC9180612; doi:10.3390/foods11111577)
Supplement: Supplementary file 1 [file foods-11-01577-s001.zip › foods-1696779-supplementary.pdf]

Table S1 *C. botulinum* and *E. coli* strains and whole genome sequences used in this work. *Strains harboring the toxin cluster.

Strain name	Isolated from	Source
C. botulinum		
KI2	Meat (Chicken)	Isolated in this work
ME2.2	Faeces (horse)	Isolated in this work
CH2	Dried mushrooms	Isolated in this work
ZS1	Seaweed sample 1	Isolated in this work
ZS2	Seaweed sample 1	Isolated in this work
ZS6	Seaweed sample 1	Isolated in this work
EV3	Meat (Wild boar)	Isolated in this work
KO2	Meat (Rabbit)	Isolated in this work
RO3	Creamy cheese sample 1	Isolated in this work
RO132	Creamy cheese sample 1	Isolated in this work
MIL1	Cheese from alpine milk	Isolated in this work
VAP23	Pond sample 1	Isolated in this work
VAP33	Pond sample 2	Isolated in this work
VAP41	Pond sample 3	Isolated in this work
VAP51	Pond sample 4	Isolated in this work
VAP52	Pond sample 5	Isolated in this work
ZBS2 (ZBS3-12-089-02)	Intestine (Pike)	Robert Koch Institute
ZBS3 (ZBS3-12-111-01)	Bream	Robert Koch Institute
ZBS4 (ZBS3-12-114-02)	Lake sediment	Robert Koch Institute
ZBS5 (ZBS3-13-004-02)	Faeces (horse)	Robert Koch Institute
ZBS12 (ZBS3-14-386-02)	rumen content (cattle)	Robert Koch Institute
ZBS13 (ZBS3-14-387-02)	Faeces (cattle)	Robert Koch Institute
ZBS14 (ZBS3-14-405-01)	Duodenum (Mallard duck)	Robert Koch Institute
ZBS15 (ZBS3-14-419-04)	Biogas plant	Robert Koch Institute
ZBS17 (ZBS3-15-138-01)	Silage	Robert Koch Institute
ZBS18 (ZBS3-15-180-01)	Stomach content (human)	Robert Koch Institute
ZBS20 (ZBS3-H163-01)	Honey	Robert Koch Institute
NCTC8266Δbont::ermB*	Canned salmon (Canada)	Clauwers *et al*., 2016
NCTC11219Δbont::ermB*	Canned salmon (Alaska)	Clauwers *et al*., 2016
DSM1985		German Collection of Microorganisms and Cell Cultures, DSM
CMCC3676		Colworth Microbiology Culture Collection, CMCC, Parker *et al.* 2015
CMCC3677		Colworth Microbiology Culture Collection, CMCC, Parker *et al.* 2015
CMCC3678		Colworth Microbiology Culture Collection, CMCC, Parker *et al.* 2015
K3*	Trout (Finland)	NZ_LFQX00000000.1
K15*	Trout (Finland)	NZ_LFQW00000000.1

CDC66177*	Argentinian soil	NZ_ALYJ00000000.1
KAPB-3*	Salted whitefish (California)	NZ_JQOK00000000.1
NCTC8550*		NZ_CP010521.1
Beluga*	Fermented whale flippers (USA)	NZ_ACSC00000000.1
202F*	Marine sediment (USA)	NZ_NPMX00000000.1
Eklund 17B*	Marine sediment (USA)	NC_010674.1
Alaska E43*	Salmon eggs associated with foodborne case (Alaska)	NC_010723.1
E. coli		
DH5α		[62]
S17λpir		[63]

Table S2 Growth media and medium supplements used in this work.

**Medium**	**Composition/Brand/Cat N°**	**Reference or source**
C. botulinum		
Reinforced Clostridial Medium (RCM)	38 g/l /Oxoid/CM0149	Thermofisher Scientific (Waltham, MA, USA)
E. coli		
Lysogeny Broth (LB)	Tryptone 10 g/l /Lab M/MC005	Thermofisher Scientific (Waltham, MA, USA)
	Yeast extract 20 g/l /Oxoid/LP0021B	Thermofisher Scientific (Waltham, MA, USA)
	NaCl 5 g/l /Fisher Chemical/S271-500	Acros Organics (Geel , België)

For solid media, 15 g/l agar was added (Neogen, Lansing, MI, USA)

**Medium supplement**	**Concentration**	**Supplier/Cat N°**
Erythromycin (Em)	5 μg/ml (construction of Em resistant mutants)<br>3 μg/ml (Selective medium)	Acros organics/227330050
Gentamicin (Gm)	15 μg/ml	Fluka Biochemika/GA11897
Cycloserin (Cs)	250 μg/ml (construction of Em resistant mutants)<br>100 μg/ml (Selective medium)	Acros organics/228480010
Thiamphenicol (Tm)	15 μg/ml	Thermo Scientific/15700899
Chloramphenicol (Cm)	30 μg/ml	Acros organics/227920250
Lysozyme (Lys)	10 μg/ml (heat resistance of spores)<br>50 μg/ml (selective medium)	MP biomedicals/100834

Table S3 Sequences of the target genes for ClosTron intron insertion, and sequences of the retargeted introns. The introns insert between the red nucleotides indicated in bold.

Gene *XkdK* (targeted in strains ZBS4-VAP51-ME22), intron insertion between nucleotides 471 and 472 in sense orientation.
ATGGCAGGTGGAACATGGGAAAAACAAAATAAAATAAGAGCAGGAGCTTATGTTAATTTTAA<br>AAGTAAAAAGAATAATGATAGTAACAATAATGATAGAGGGGTAATGGCGTTACCGTTAGTATT<br>ACCTTTTGGACCAGAAAAAACAATAGTAAAAATAGATAATGAAACAGATTTGTTAGGAACAA<br>TAGGAATGGAAATAAATGAAGAAAGCACTCTAATGCTTAAAGAAGCCTTAAAGAAAGCAAA<br>AACAGTTTTATTAACAGACTTAATGAAGGTGTTAAGGCTACTAAGGTGCTAGGAGAATTAACA<br>GTAACATCAAAATGGAGTGGAAGTAAAGGAAATGATATAAGAATTCAAATTCAAACCAATGT<br>AAATGATGAAGGTAAATTTGATGTAATTACATTCTTAGAATATACAAAATTAGATACACAAAC<br>TGTAAAAAATATTGATGAGTTGGTATCTAATG**CA**CTTGTAGACTTTAAAGGATCAGGTGAACTT<br>ACTTTATCTGCTGGAGTAAAGCTAGAGGGTGGAGAAGATAAACCTATTACAGGAAAGGATTAT<br>GTAGATTTTTTAGCAGAATTAGAATTATTTGATTTCAATACTGTAGCAATACCGTATGATGAGT<br>CAGATACTAAGTTAGTTGTAAAAGAGTTTATTAAGAGGTTAAGAGAATATGAAGGAAGAAAA<br>GTTCAAGCTGTTTTACCAAACTTTGCAGAAGCAGACTATGAAGGGATTATATCTATAAAGAAT<br>GGTGTGTATCTTAAAGATAATGTACATGTAACTAATGTACAAGCAACAGCATATGTAGCAGCA<br>TTAACAGCAGGATCAGGTTATGCTAATTCTAATACTTATGCTTTGTATGAAGGTGCAAGCAATG<br>TTGATGTTAGATATGCAGATAGTGAAATAAAAGAGATAATCAAAAAAGGTGAAATAATATTTA<br>TTAATAATAATCAACAAGTTTTAATTGAACAAGATATTAACACATTAAAGACTTTTACAGAGG<br>ATAAAAAGTCTGACTTTAGAAAAAACAGAGTTATAAGAGTTTTAGATGGAATAAATGACAAG<br>ATTAAATATAAGTGGGAAGAATCTTATATTGGCAAAGTAAGCAATAATGAGGATGGAAGAAA<br>CTTATTTAAGAAAGATATATTAAATATTTTAGAAACATTACAAGGACAAGGAGCATTAGAAAA<br>TGTAGTTGTAGAGGATATTGAGGTACTTAAAGAAATTCTAATGATTCTATTGTTGTAAATGTTA<br>ATGCGCAACCAGTAGATAGTATGGAAAAAATATATATGACTGTATTTATATAA
Intron sequence targeted towards *XkdK* (471-472s)
TTATCCTTAGTATCCAATGCAGTGCGCCCAGATAGGGTGTTAAGTCAAGTAGTTTAAGGTACTA<br>CTCTGTAAGATAACACAGAAAACAGCCAACCTAACCGAAAAGCGAAAGCTGATACGGGAAC<br>AGAGCACGGTTGGAAAGCGATGAGTTACCTAAAGACAATCGGGTACGACTGAGTCGCAATGT<br>TAATCAGATATAAGGTATAAGTTGTGTTTACTGAACGCAAGTTTCTAATTTCGATTGATACTCG<br>ATAGAGGAAAGTGTCTGAAACCTCTAGTACAAAGAAAGGTAAGTTAAGTGCATTGACT
Gene *SPP1* (targeted in strain ZBS3), intron insertion between nucleotides 105 and 106 in sense orientation
ATGTTGGGAGTTAAAAGTATATGGAATAAACTTAAGAAAGGAGTGAAAGCAGGTATGGCAGC<br>AGCACAAGTTAAAAGTATTATTGATGATGAGCAGATTGTTGG**AA**TGATAACTGAGTTTAATAT<br>GTCACAAAAAAGAAAACTTATGATTATAGGTTCTAAATATTATGAGGTTGAAAATGATATTTTT<br>AAAAGAAAACTAACAAAAGTAGTTGATGGTGAAACAATAGAAGAAACTTATAAGGCTAATA<br>ATAAACTAGCACATGCTAAGTATAAAAACATAGTTGACGAAAAGGTTGCATACTTGCTTTCAA<br>GACCCTATTCTTTAAATTGTGATGATAAGCAGTATATAGAAAAAATCAAGGATTCGCTAGGGA<br>AGCACTTTCAATATAAGTTGTCAGGATTAGGATATGAGGCAAGCAACAAAGGTATTTCATGGT<br>TACAGCCTTATATAAATGAACAGGGTAAATTTAGTACTATGATTATACCTTCAGAGCAATGTAT<br>CCCTATCTGGAAAGACAATAGCCATATGGAGTTAAGTGGCATGATAAGAATATATGAAACAA<br>CTTACTGGGTTGGATCTACTAAGAAAACTGATACTAATGTTGAGGTTTGGAGCAAAGATGGCG<br>TTGTTTATTATAAATTAGATGATAAGAAATTAATATATGATTATGGCAAATCTAATGATATAGA<br>CAATGGCGGCCCAATAGCACATTTTAAAAGAGATGATGAGTGGATTTCATGGGGAAAGGTTCC<br>TTTCATACCGTTTAAAAATAACAGAGTAGAAATGCCTGATATTAAGTTTGTTAAATCTCTACTA

GATGAGTATGATAAGTCGAGATCAGAAGCAGCTAATTATGTGGAAGATGTAAAGAATTTAATA
TTTATTTTAAAGGGTTATGGTGGAGAAGATATTAAGGAGTTTATGAAGCATCTTAATGAAGATA
GAGCAATTCCAATTGATGACCCACAGGATGGTGGAGTTGATGCTTTAACTCCTACAATGGACA
TAACAGCTTTAAGAGAACATTATGAACAACTTAAAAGGGATTTAACAGAAGATGGACAAAGT
ATTAATAAAGATTTAGATAAGTTTGGTAGTGCTCCAAGTGGCGTTGCTCTTAAGTTTATGTATG
CAGGACTAGATCTAAAAAGTAATGCATTAGAGGTTGAATTTAAAATGGGTTTTGAAAGTTTATT
ATATTTTATAAATATATACTTAGGTGAAACAAATCAAGGAAATTATAATAGTGATACAATTAA
TCTAGATATCGAGTTTAATAGGGATATGGAAATTAACGAAAGTGAAATTATTGATAACTGTAG
TAAATCAAAAGGCATTGTCAGCGACGAATTGATATTAGCTAATCACCCATGGGTTAAAGACAT
AGAAAAGGAAAATGAGGCATTAGATAAGCAGACTAAAGCTAGTTTGCCATTTAAAGATAGAG
TTCCTATAAATGAGGGTGATTCTATTGAATAA

Intron sequence targeted towards *SPP1* (105-106s)

TTATCCTTACAGATCGTTGGAGTGCGCCCAGATAGGGTGTTAAGTCAAGTAGTTTAAGGTACTA
CTCTGTAAGATAACACAGAAAACAGCCAACCTAACCGAAAAGCGAAAGCTGATACGGGAAC
AGAGCACGGTTGGAAAGCGATGAGTTACCTAAAGACAATCGGGTACGACTGAGTCGCAATGT
TAATCAGATATAAGGTATAAGTTGTGTTTACTGAACGCAAGTTTCTAATTTCGGTTATCTGTCGA
TAGAGGAAAGTGTCTGAAACCTCTAGTACAAAGAAAGGTAAGTTAATTCCAACGACT

Gene *TTMP* (targeted in strain CH2), intron insertion between nucleotides 523 and 524 in sense orientation

ATGTCGATAAATGTAGGATCGGCCATTGCATATCTAACCTTAGATAGATCAAGCTTTACAAATG
GAATCAAATCAGCGGGAGAGGATTTGAAAAACTTTGTTAGTGGATCAAATAGTGCAGAAGATT
CCATAAAGTTATTGAGAAATTCAATGAAATCTGTAGGATCTGCAATGGCCATGCCTTCCACGA
AAGCTGGAGACTTCTTAGCGGTAGCAACAAACACCGCTATGGGTTTTGAGGAACAAATGTCTA
AAGTTAAGGCAATAAGTGGAGCTACTGGCGATGACATGGTAAAATTATCAACTCTATCTAGGG
ATATGGGAGTCAGTACAAAATTTAGCGCCAAGGAGAGTGGCAAGGCCCTAGAATATATGGCG
ATGGCGGGTTGGAAAACCGATCAAATGATGGAAGGATTGCCGGGCATTTTAAACTTGGCAGCC
GCAGCGGGTGGGGATCTAGGTAAAACTTCCGATATTGTAACTGATGCGTTAACAGGATTCGGA
TTAACTGCCAAGGATAGT**GG**CCATTTCGCTGATGTGTTGGCATCTGCCAGTAGTAATGCAAAG
ACGAATGTTAGTTTACTGGGTGAATCATTTAAATATGTTGCACCAGTAGCCGGTGCGTTAAAGT
ATTCAGTCGAAGATACATCGTTAGCATTGGGATTGATGGCTAACTCAGGAATCAAGGGATCAA
GTAGTGGTACTGCACTTAGGGCCGCATTGACGAATATGGTTGAACCTACAGATAACATGGCAA
ATGTGATGGATCAATATGGTATTAGTCTTACTAATACCGATGGTAGCATGAAGTCATTAAAAG
AAGTTATGGATACCTTGAGGAATAAGATGGGTGGCCTCGATGAGCAAACCAAGAAATCAGCA
GCCGCAACACTTTTCGGAAAAGAGGCAATGTCCGGTATGCTTAGTGTTATTAATGCTAGTGATG
CTGATTATAACAAGTTATATGAAAATTTAACTAATGCCGATGGTGCAGCAAAAAAAACAGCTG
ATACAATGCAAGATAATTTAAAAGGAAAGTTGACAACCTTAAATAGTGCATTTGAGGAATTAC
AAATATCATTAGCAACCGCAGTTGTACCAATGTTAAATAAATTAGTTTCAATATTAATCACATT
AATTAATAAATTTAACGCTTTACCACAGCCAGTAAAATCAGCCATTGGAATGATAATTGGTGG
TATTGCTTTGTTTAGTCCAGCATTTTTGATATTAGGAAAATTAGTTAACACCATTGGAACCGTGG
TCGGTGTGTTTAGTAAACTTAAGACTGCTGCAAATATTTTTAAACTGTTACCTATGATAATGAGT
CCACCAGTTTCAGGGATTATATTAATTATTGGAGCCATTATACTTGTAGTATATGAGGTTATAA
AACATTGGGATAACTTAAAATCATATTTTGGAAACTTTTGGGCATGGGTTAAAAATATCTTTAG
TAGTTTTTGGGAGTGGCTTAAAACATTCTTTAGTAAATGGGGGCCAACGATCTTGACCATTATC
GCGCCATTTTTAGGAATACCACTATTAATAATTCAACACTGGGATAAAGTAAAAGAGGCCTTT
ACATTTTTGTTTGAACATTTAAAAACTATATGTACTAAAATCGGAACATTTTTCAGTAATGCTAT
AGAATTTTGGAAAGGTATTTGGAATGGTTTTAAAGACTTCATGGGAAGTATAGGCGATTGGATT
GTTGATGGTTTATTAGGTGGACTAAAAAGGGGTATGGGTAAAATAAAAGAGGGTGCATCAAAT

ATGGCTAATGGTGTAAAAGATACATTTAAAAAACTACTGGGTATTAACTCACCATCGCGAGTA<br>TTTAAAGGATATGGTGTTAACATTGGCGAGGGTTTAATTGAGGGAATGGATTCCCACGAATCA<br>GCCATTAACGGTAAGTTTGAAGGTGTTTCGAACAAGATTAAGAAACTTGGAAATATTAAACCG<br>AATTTTAATTTAAACAATACTGGTTTAAGTGGTGATTATATTGGATCAAGTAACATTGCAAATT<br>CAAGTGCCAAACAACTTAATTTTACACCTAATATTAATATGCAAGTTCATATTGCTGATACTGG<br>AGCAAAAGGCACGGCGCAGCTTACAAGTGAACTTAAGAGCATGGCCCAAAATGCATTTAAAA<br>ATAGTATGGTTAATGAATTCATGGCAGATGCTTTAAGACTATAG
Intron sequence targeted towards *TTMP* (523-524s)
TTATCCTTACCAAGCATAGTGGTGCGCCCAGATAGGGTGTTAAGTCAAGTAGTTTAAGGTACTA<br>CTCTGTAAGATAACACAGAAAACAGCCAACCTAACCGAAAAGCGAAAGCTGATACGGGAAC<br>AGAGCACGGTTGGAAAGCGATGAGTTACCTAAAGACAATCGGGTACGACTGAGTCGCAATGT<br>TAATCAGATATAAGGTATAAGTTGTGTTTACTGAACGCAAGTTTCTAATTTCGGTTCTTGGTCGA<br>TAGAGGAAAGTGTCTGAAACCTCTAGTACAAAGAAAGGTAAGTTAGCCACTATGACT

Days to growth (OD_{620} >0,15) of 1 or more replicates

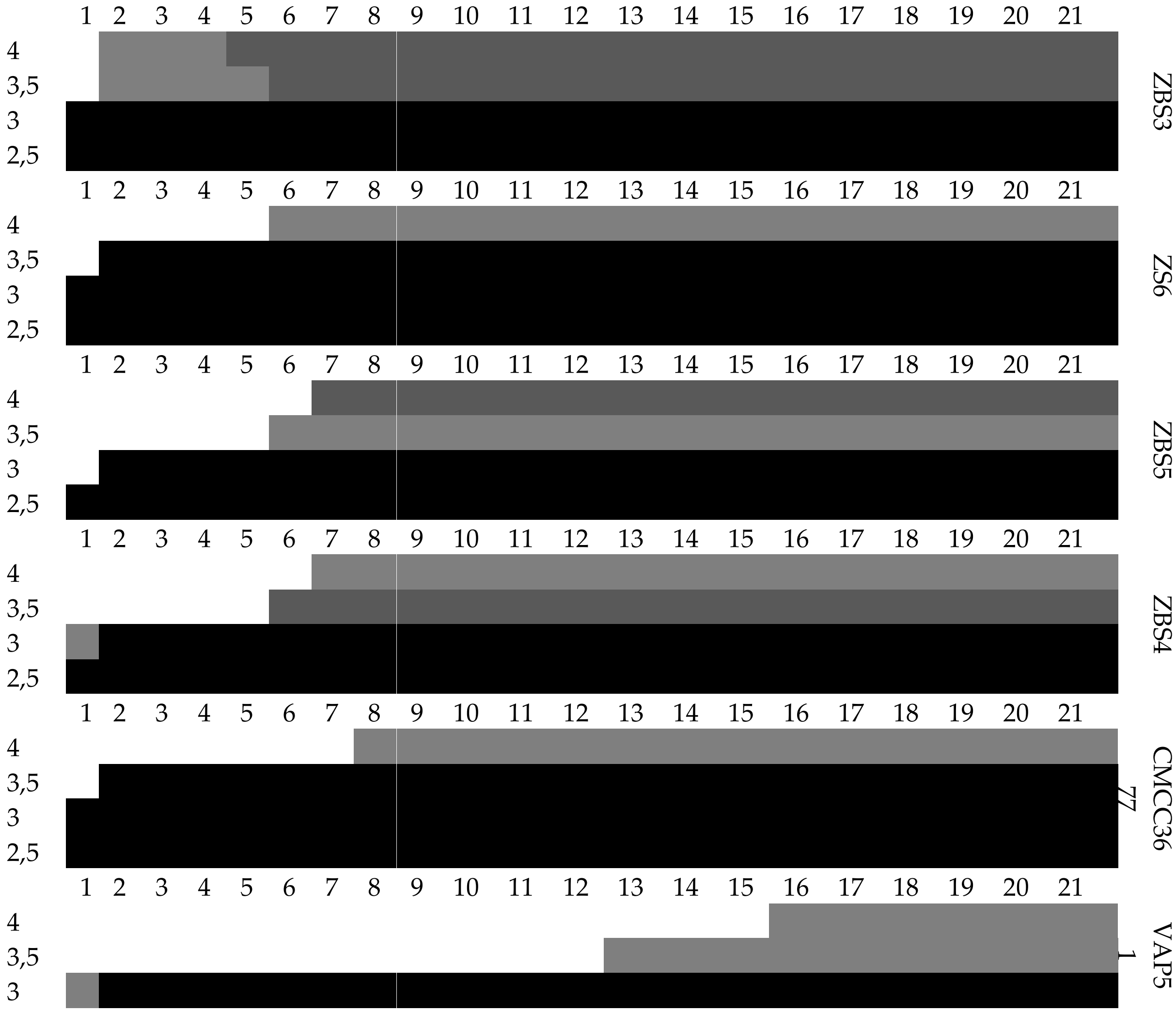

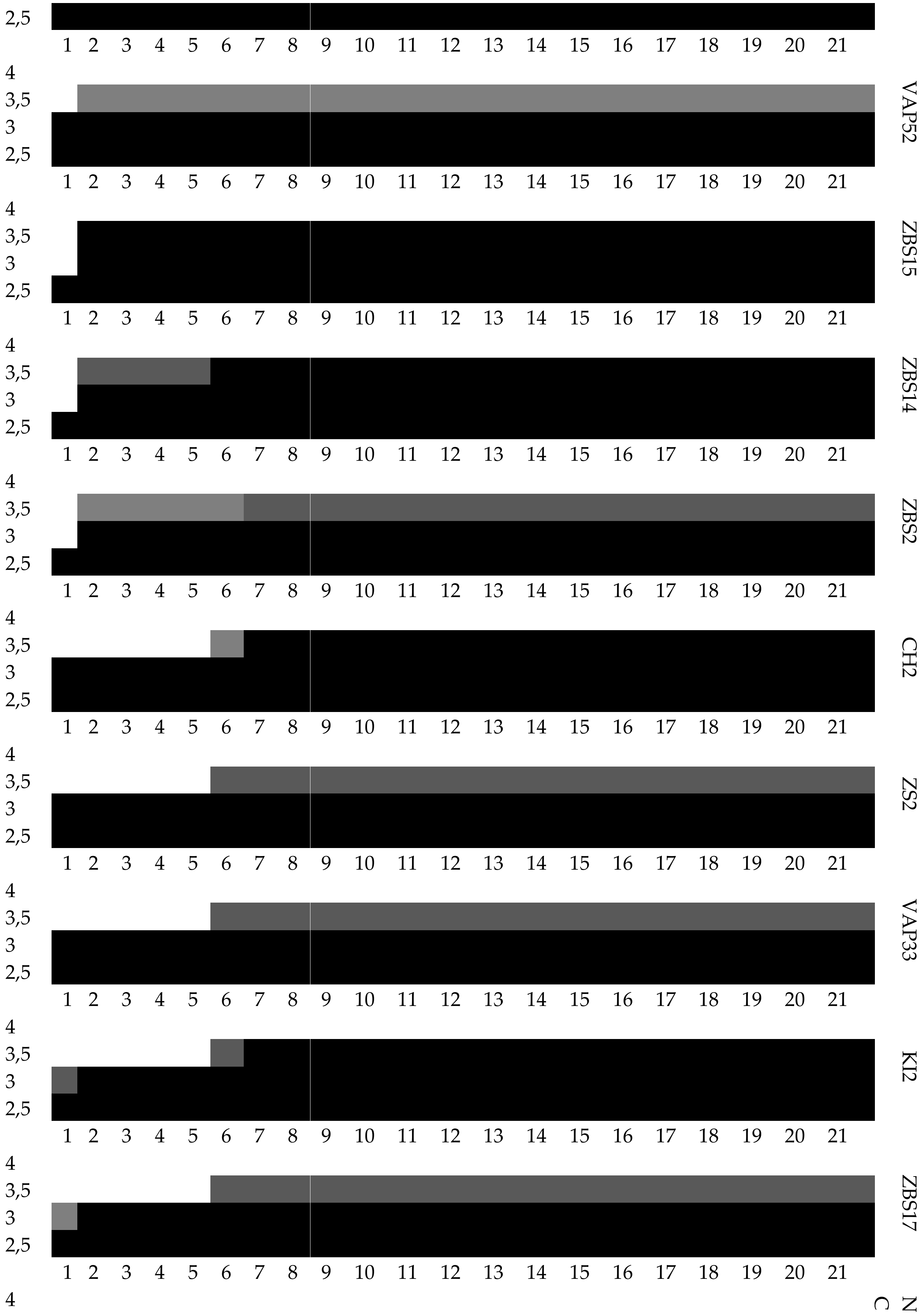

VAP52
ZBS15
ZBS14
ZBS2
CH2
ZS2
VAP33
KI2
ZBS17
N
C
1 2 3 4 5 6 7 8 9 10 11 12 13 14 15 16 17 18 19 20 21
2,5
3
3,5
4

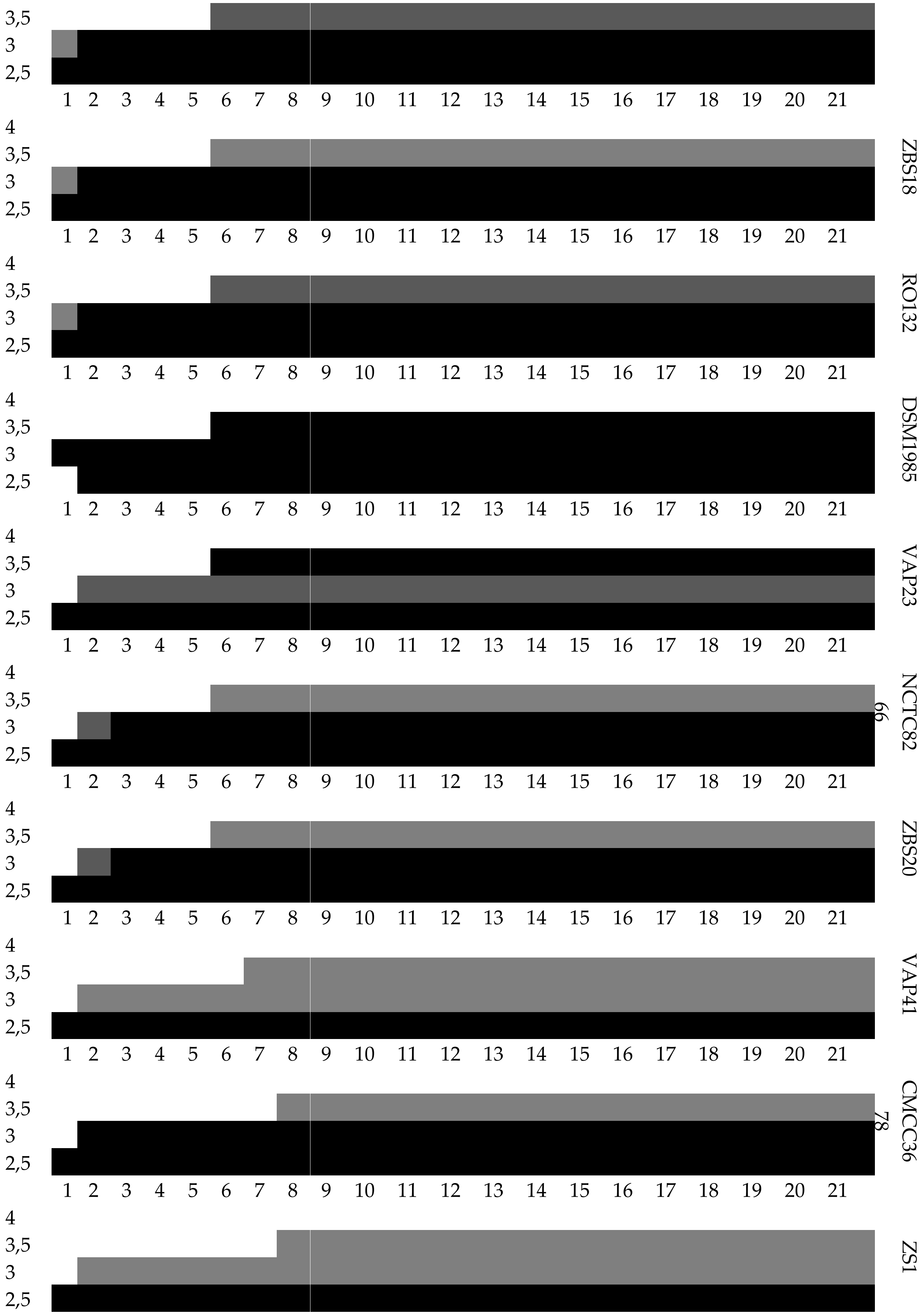
4
3,5
3
2,5
1 2 3 4 5 6 7 8 9 10 11 12 13 14 15 16 17 18 19 20 21
ZBS18
RO132
DSM1985
VAP23
NCTC8266
ZBS20
VAP41
CMCC3678
ZS1

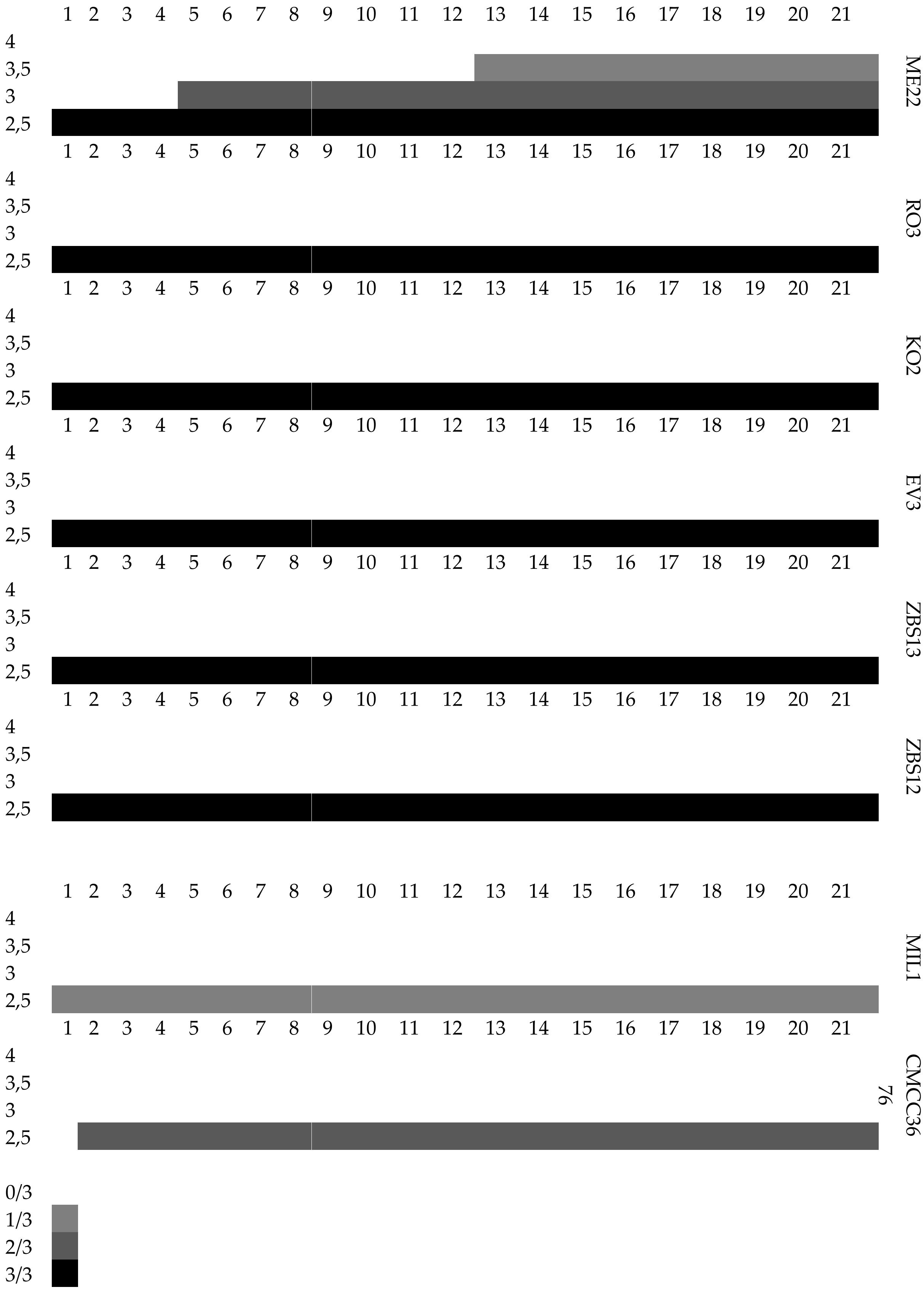
1 2 3 4 5 6 7 8 9 10 11 12 13 14 15 16 17 18 19 20 21
4
3,5
3
2,5
ME22
RO3
KO2
EV3
ZBS13
ZBS12
MIL1
CMCC36
0/3
1/3
2/3
3/3

Figure S1 Salt tolerance of different nontoxic isolates, NCTC8266Δbont::ermB and NCTC11219Δbont::ermB in liquid RCM medium supplemented with different amounts of NaCl. Growth at 30°C in 2.5%, 3.0%, 3.5%, 4.0% and 4.5% NaCl was studied for 21 days for three replicates per strain. Growth of all replicates is indicated. None of the strains grew at 4.5% NaCl.

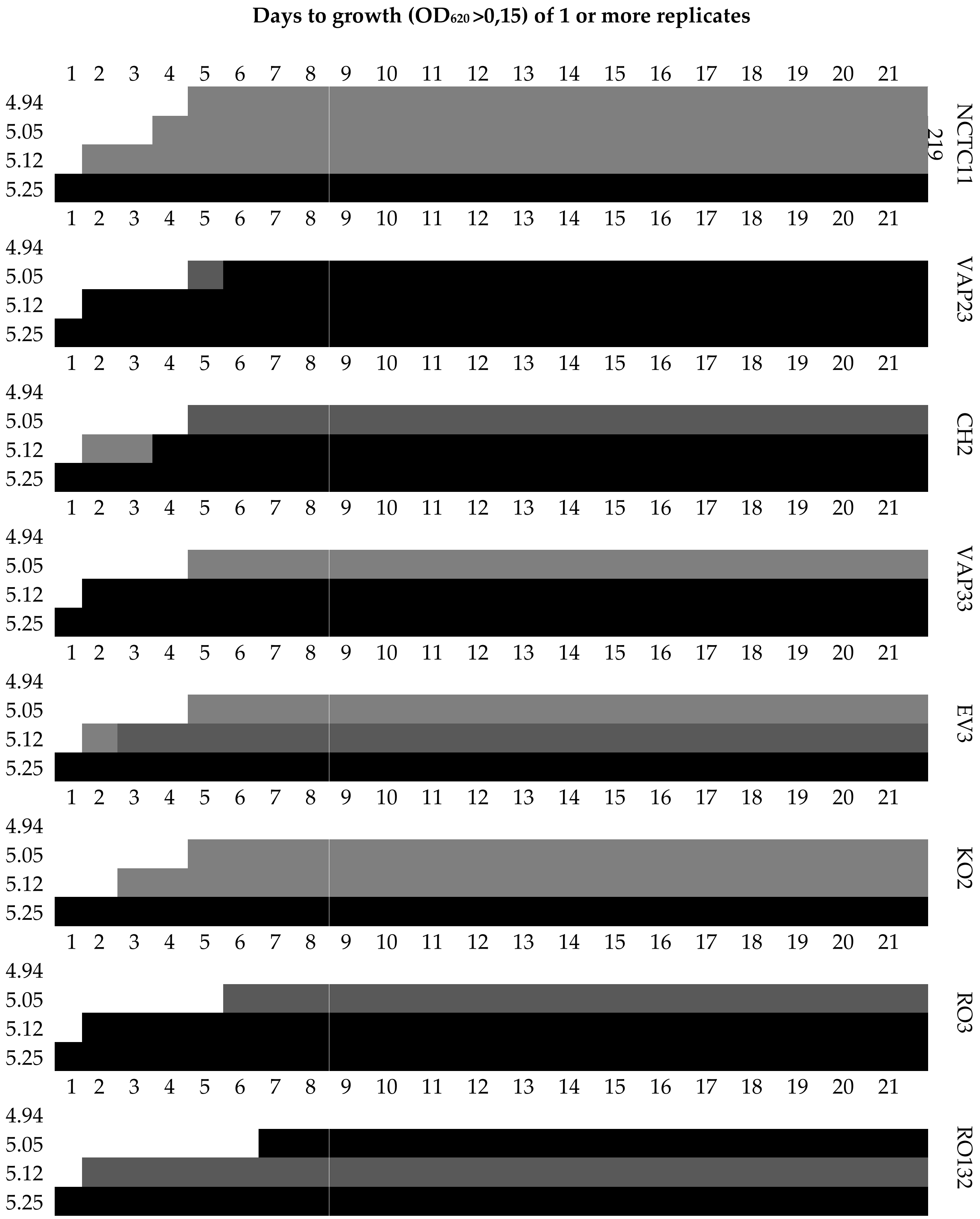

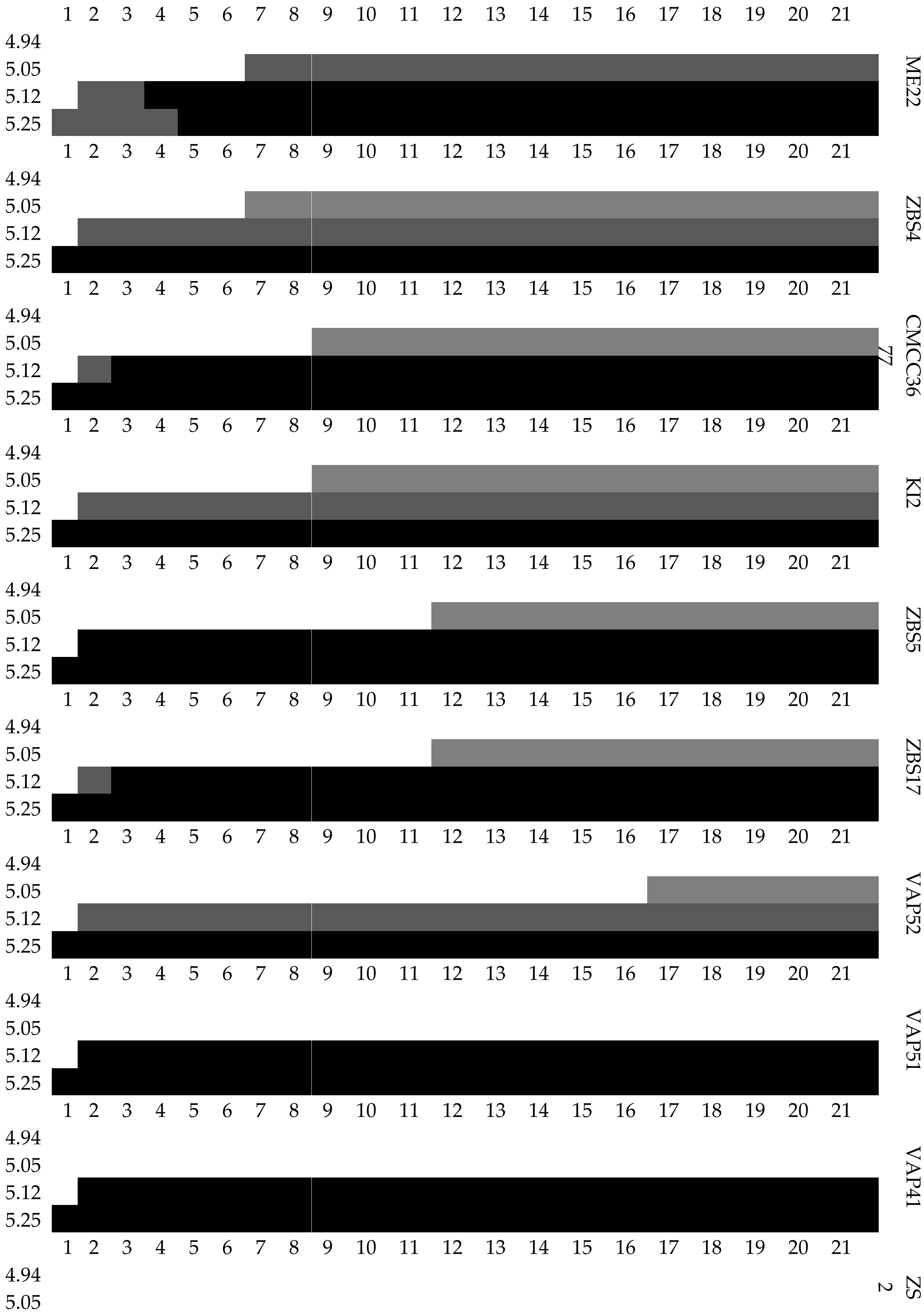
1 2 3 4 5 6 7 8 9 10 11 12 13 14 15 16 17 18 19 20 21
4.94
5.05
5.12
5.25
ME22
ZBS4
CMCC3677
KI2
ZBS5
ZBS17
VAP52
VAP51
VAP41
ZS2

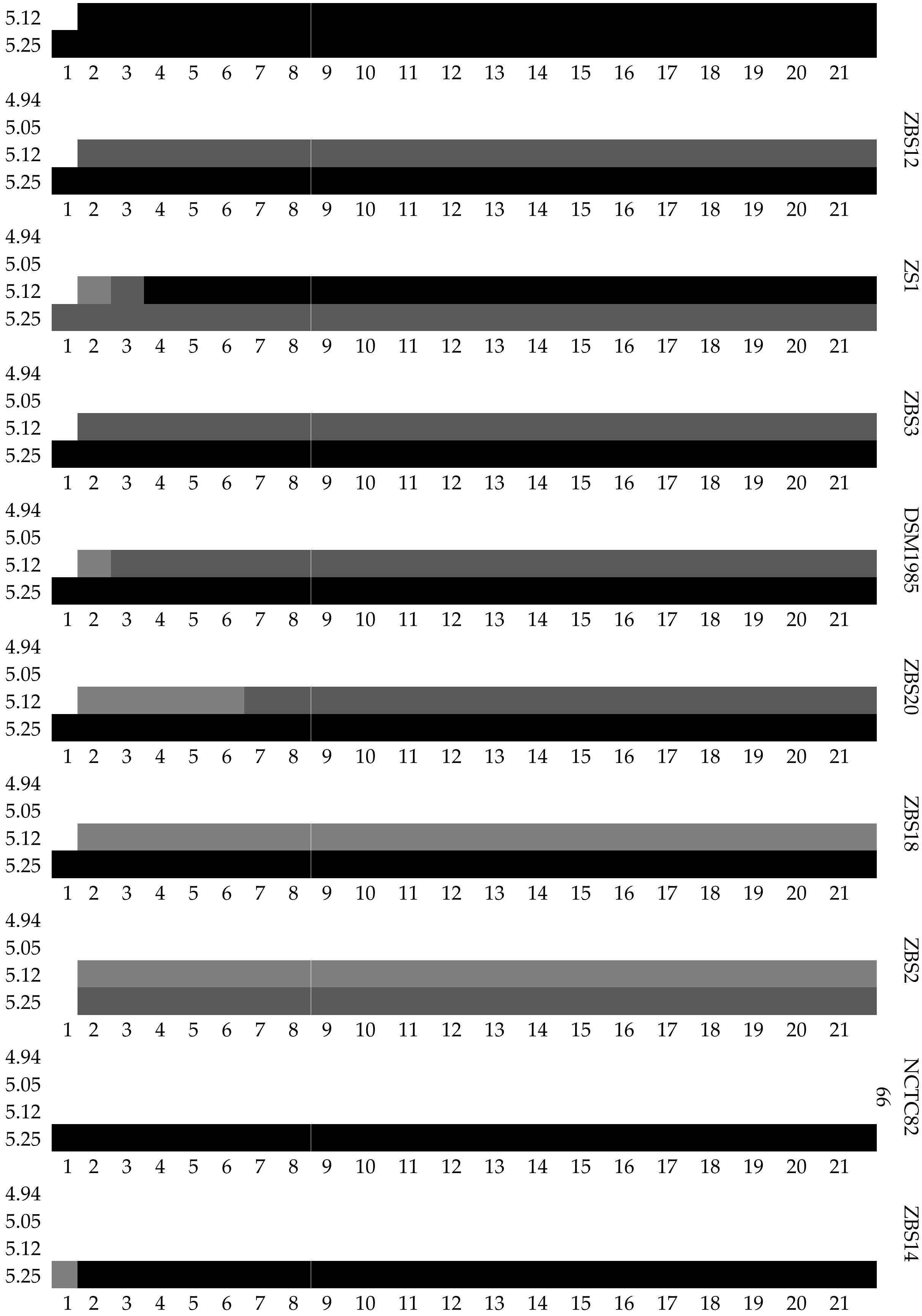
4.94
5.05
5.12
5.25
1 2 3 4 5 6 7 8 9 10 11 12 13 14 15 16 17 18 19 20 21
ZBS12
ZS1
ZBS3
DSM1985
ZBS20
ZBS18
ZBS2
NCTC82
ZBS14

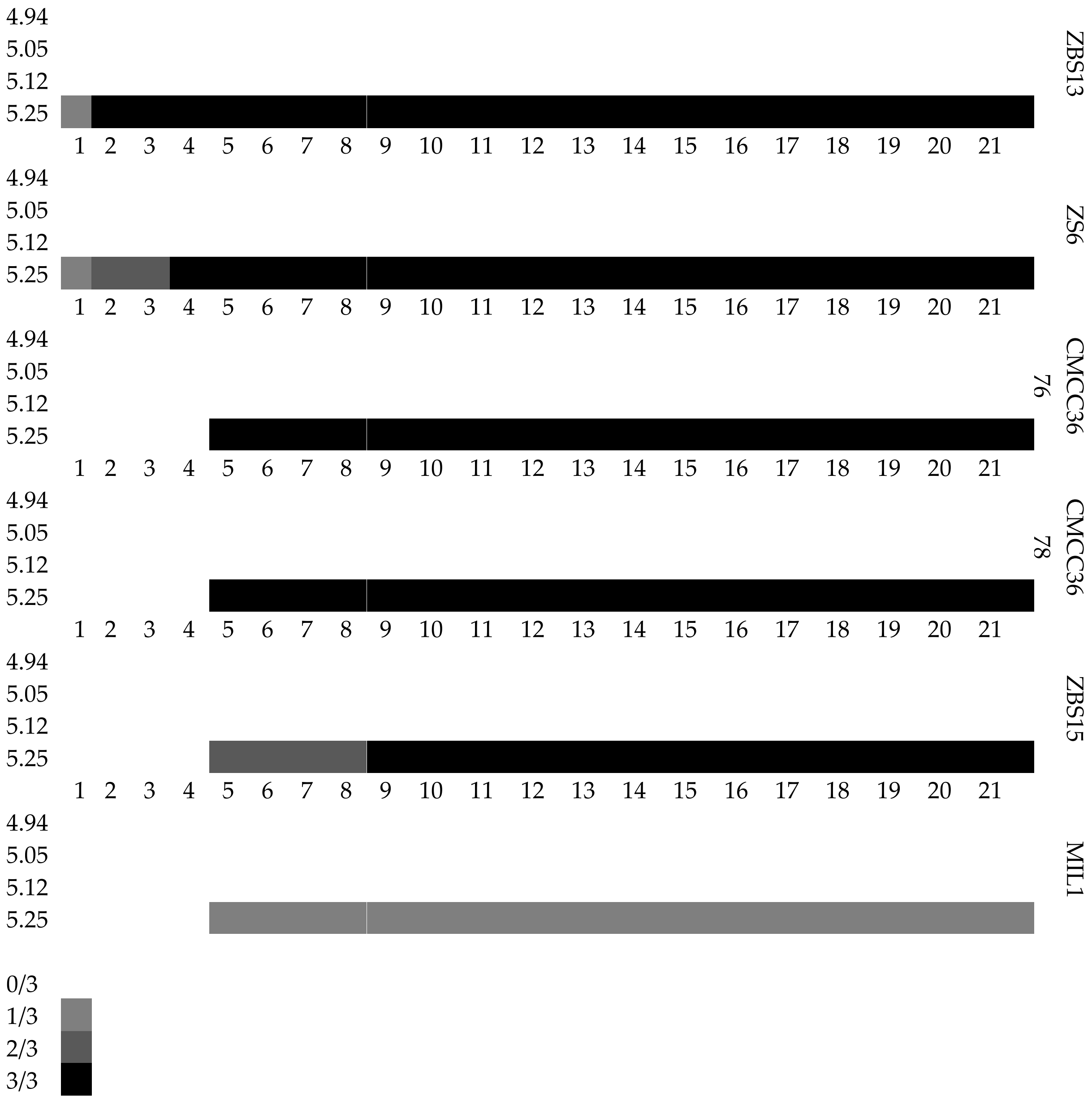

Figure S2 Acid tolerance of different nontoxic isolates, NCTC8266Δbont::ermB and NCTC11219Δbont::ermB in liquid RCM medium supplemented with 1 M HCl (pH values; 5.25, 5.12, 5.05 and 4.95). Growth was studied for 21 days for three replicates per strain. Growth of all replicates is indicated.

Days to colony formation (>1 mm) of 1 or more replicates

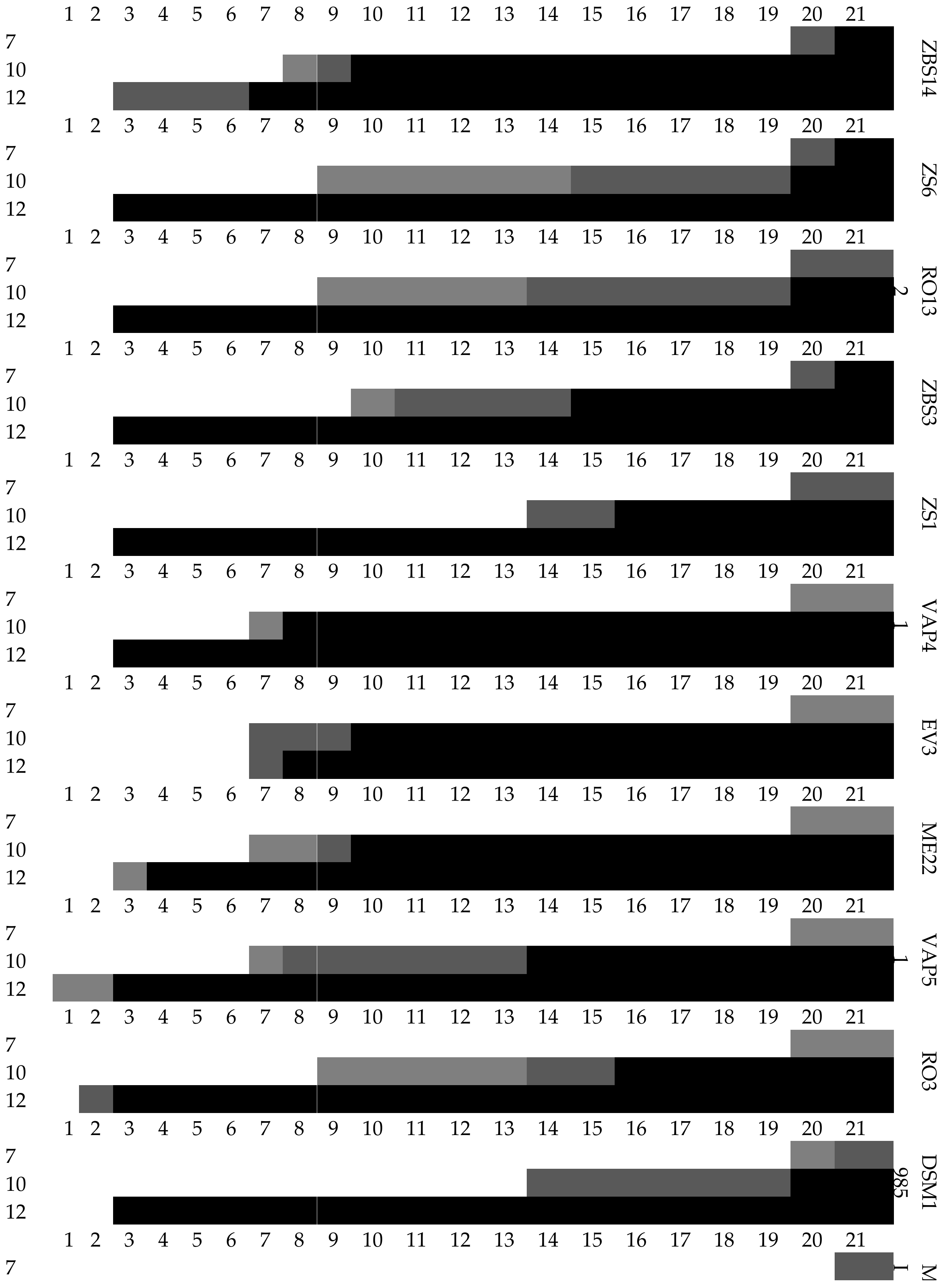

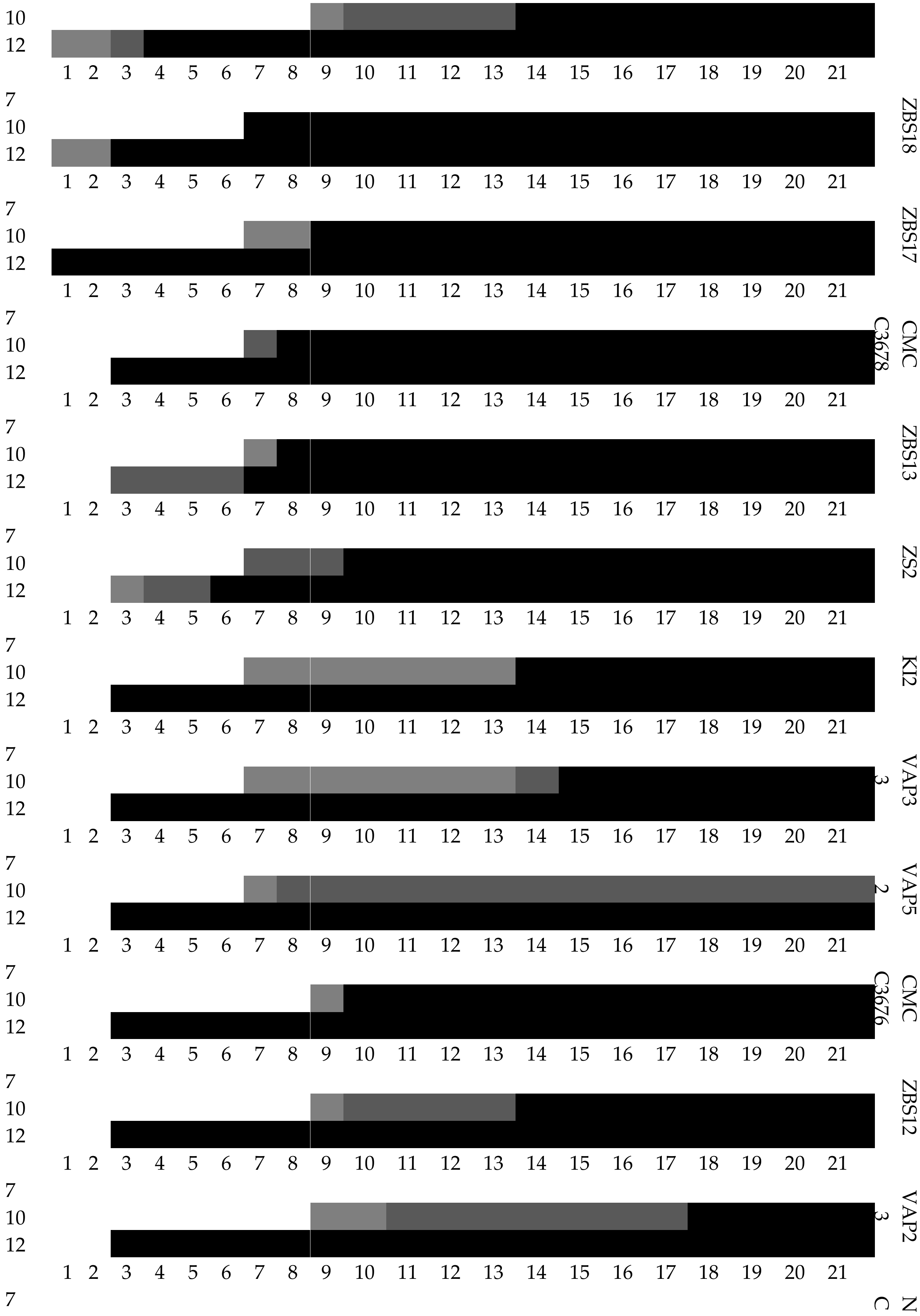

ZBS18
ZBS17
CMC C3678
ZBS13
ZS2
KI2
VAP33
VAP52
CMC C3676
ZBS12
VAP23
NC
7
10
12
1 2 3 4 5 6 7 8 9 10 11 12 13 14 15 16 17 18 19 20 21

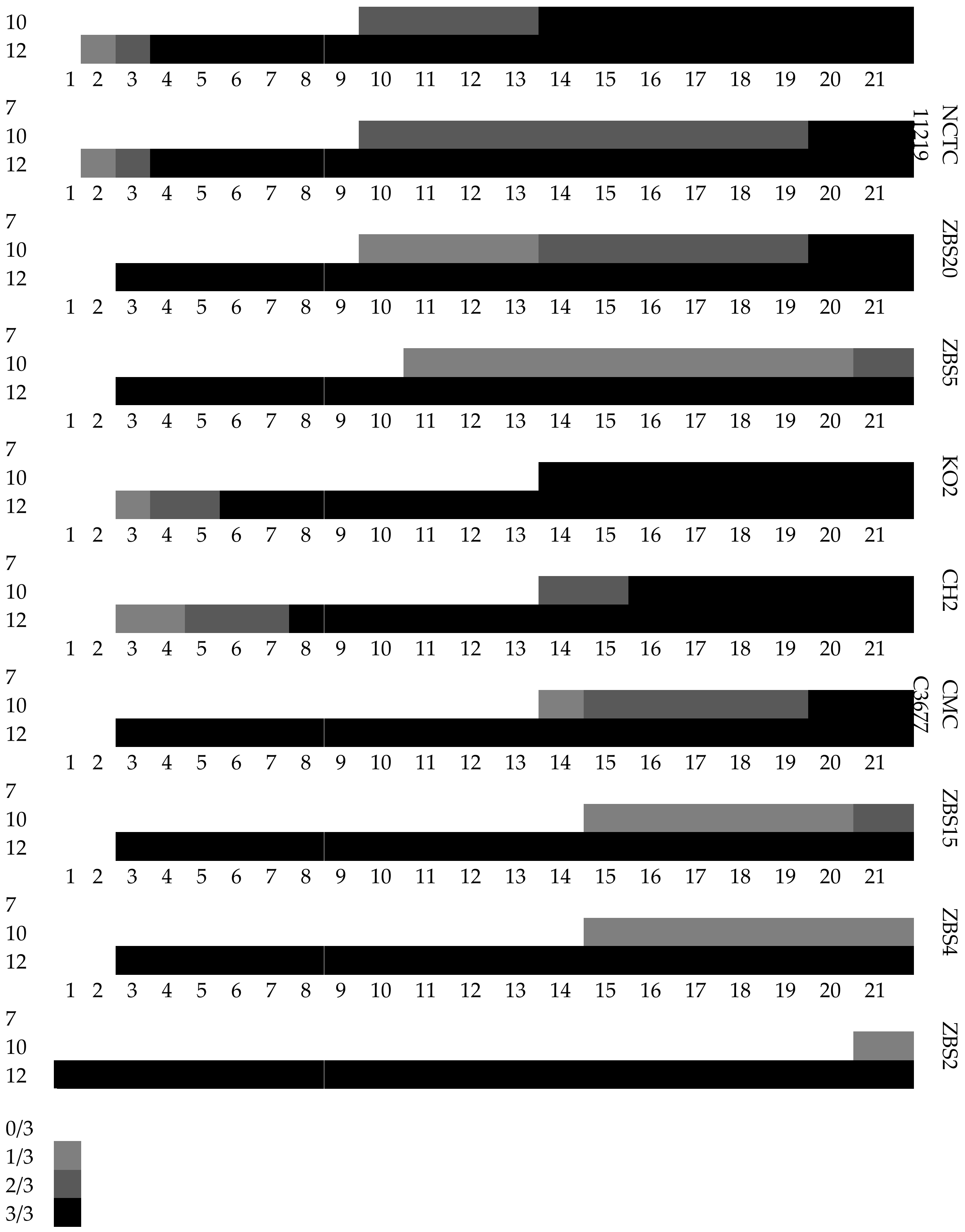

Figure S3 Low temperature tolerance of different nontoxic isolates, NCTC8266Δbont::ermB and NCTC11219Δbont::ermB on solid RCM medium incubated at different temperatures (7°C, 10°C and 12°C). Growth was studied for 21 days for three replicates per strain. Growth of all replicates is indicated.

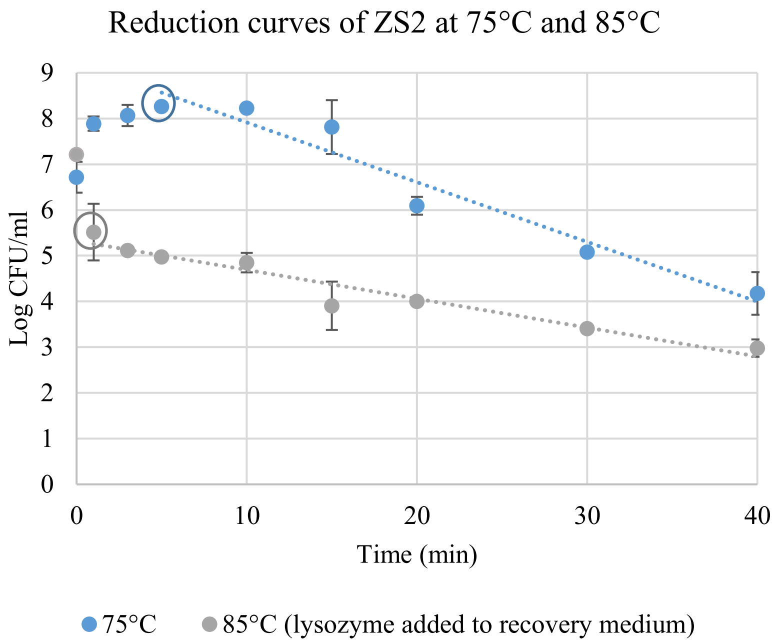
Reduction curves of ZS2 at 75°C and 85°C
Log CFU/ml
0
1
2
3
4
5
6
7
8
9
0
10
20
30
40
Time (min)
75°C
85°C (lysozyme added to recovery medium)

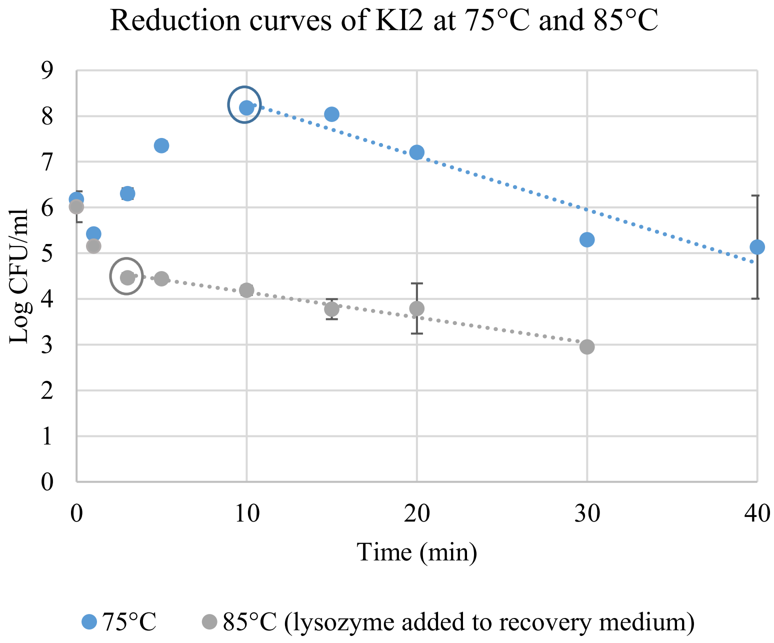
Reduction curves of KI2 at 75°C and 85°C
Log CFU/ml
0
1
2
3
4
5
6
7
8
9
0
10
20
30
40
Time (min)
75°C
85°C (lysozyme added to recovery medium)

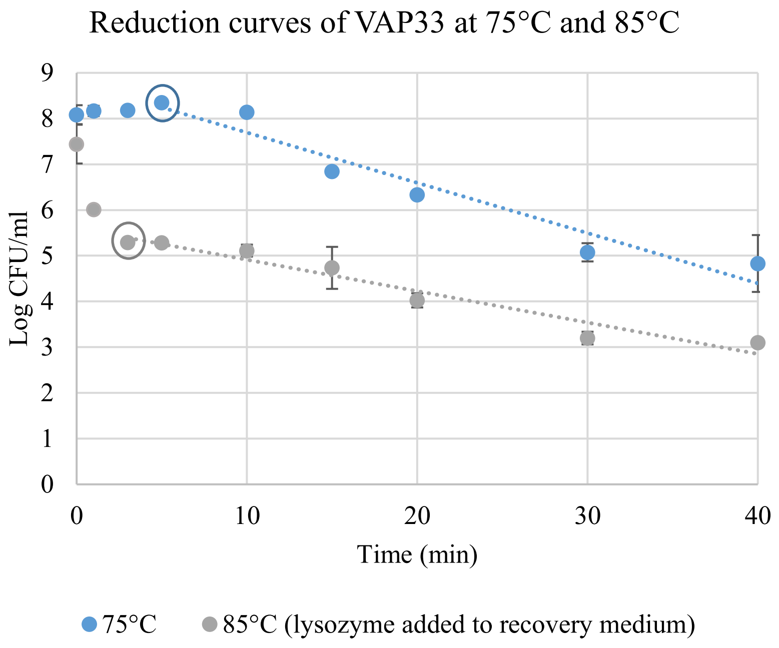
Reduction curves of VAP33 at 75°C and 85°C
Log CFU/ml
Time (min)
0
10
20
30
40
0
1
2
3
4
5
6
7
8
9
75°C
85°C (lysozyme added to recovery medium)

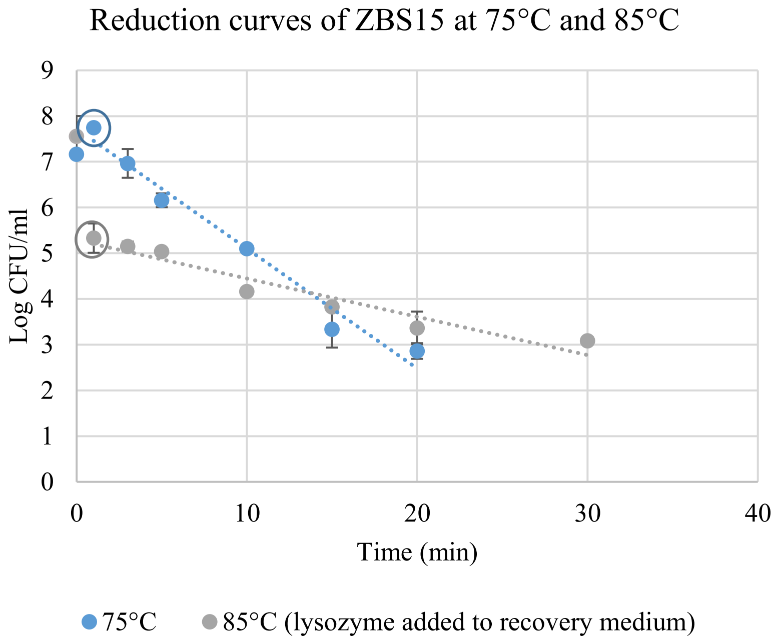
Reduction curves of ZBS15 at 75°C and 85°C
Log CFU/ml
Time (min)
0
10
20
30
40
0
1
2
3
4
5
6
7
8
9
75°C
85°C (lysozyme added to recovery medium)

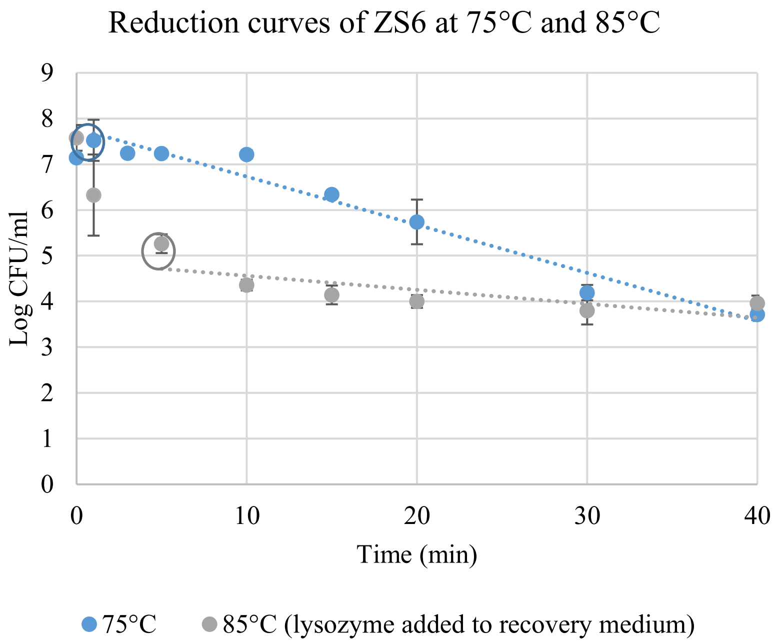

Reduction curves of ZS6 at 75°C and 85°C
Log CFU/ml
0
1
2
3
4
5
6
7
8
9
0
10
20
30
40
Time (min)
75°C
85°C (lysozyme added to recovery medium)

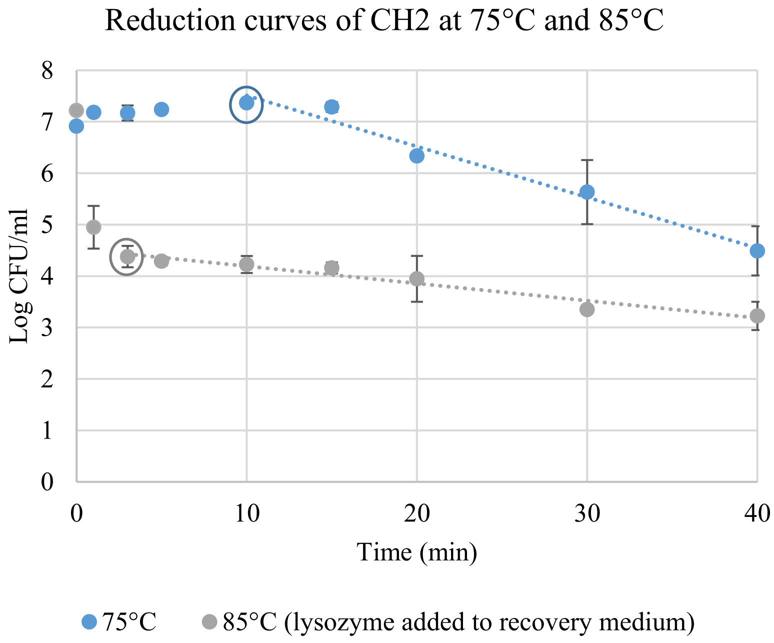

Reduction curves of CH2 at 75°C and 85°C
Log CFU/ml
0
1
2
3
4
5
6
7
8
0
10
20
30
40
Time (min)
75°C
85°C (lysozyme added to recovery medium)

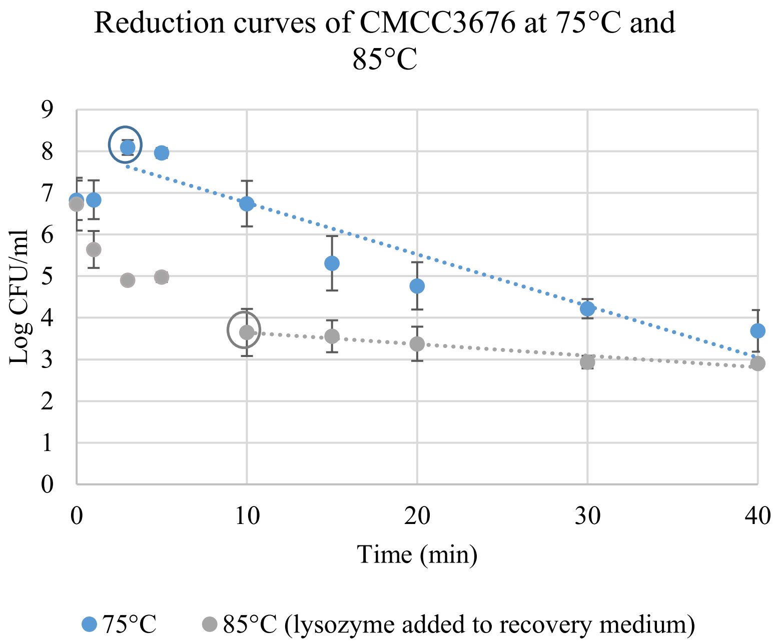

Reduction curves of CMCC3676 at 75°C and 85°C
Log CFU/ml
9
8
7
6
5
4
3
2
1
0
0
10
20
30
40
Time (min)
75°C
85°C (lysozyme added to recovery medium)

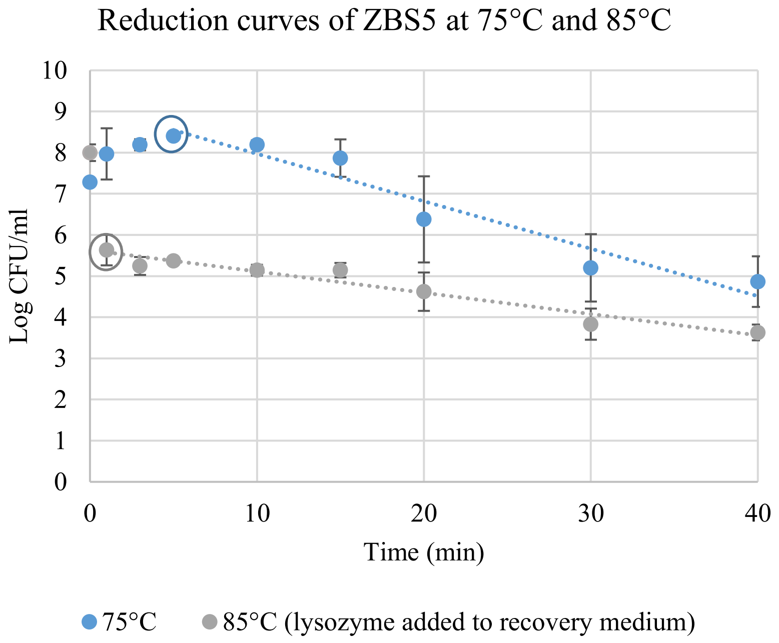

Reduction curves of ZBS5 at 75°C and 85°C
Log CFU/ml
10
9
8
7
6
5
4
3
2
1
0
0
10
20
30
40
Time (min)
75°C
85°C (lysozyme added to recovery medium)

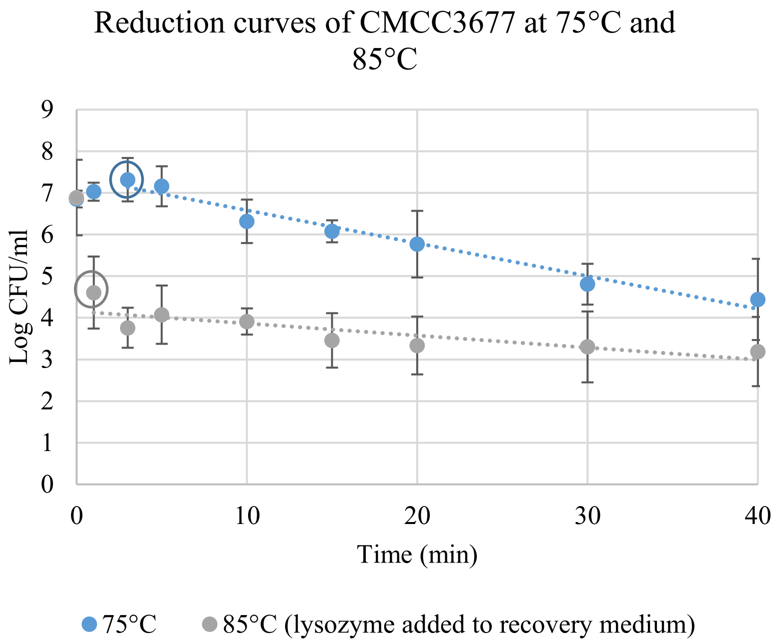

Reduction curves of CMCC3677 at 75°C and 85°C
Log CFU/ml
Time (min)
75°C
85°C (lysozyme added to recovery medium)

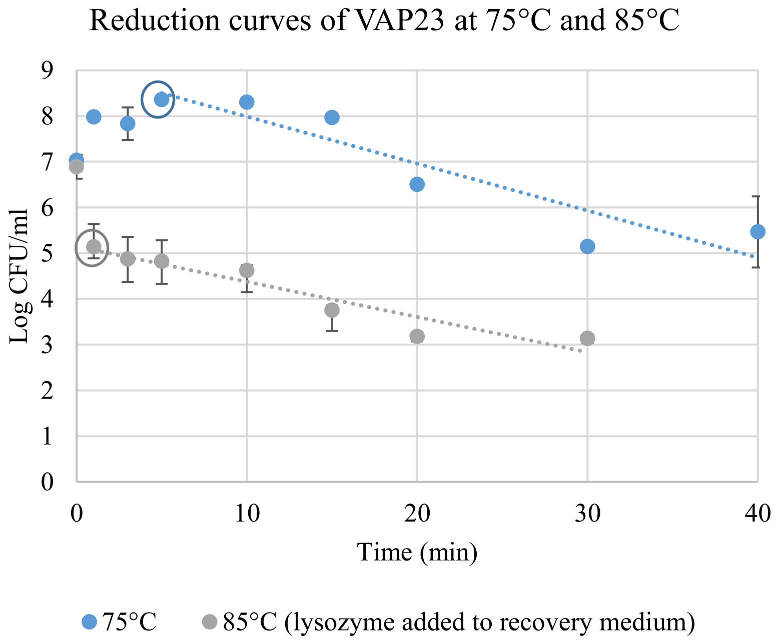

Reduction curves of VAP23 at 75°C and 85°C
Log CFU/ml
Time (min)
75°C
85°C (lysozyme added to recovery medium)

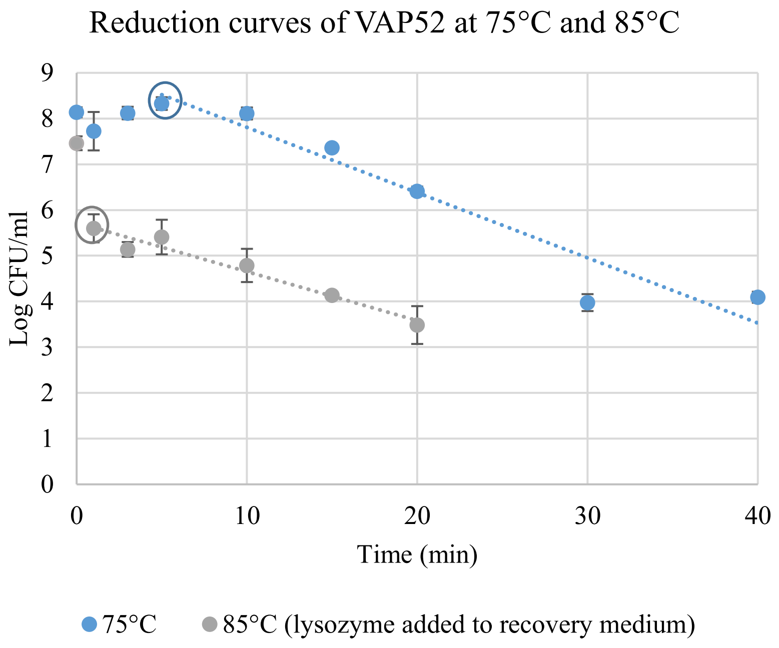

Reduction curves of VAP52 at 75°C and 85°C
Log CFU/ml
0
1
2
3
4
5
6
7
8
9
0
10
20
30
40
Time (min)
75°C
85°C (lysozyme added to recovery medium)

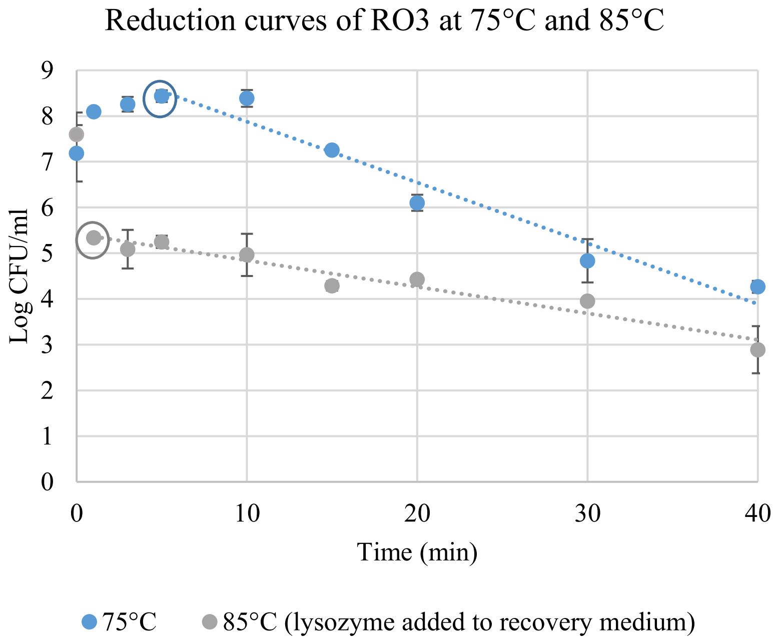

Reduction curves of RO3 at 75°C and 85°C
Log CFU/ml
0
1
2
3
4
5
6
7
8
9
0
10
20
30
40
Time (min)
75°C
85°C (lysozyme added to recovery medium)

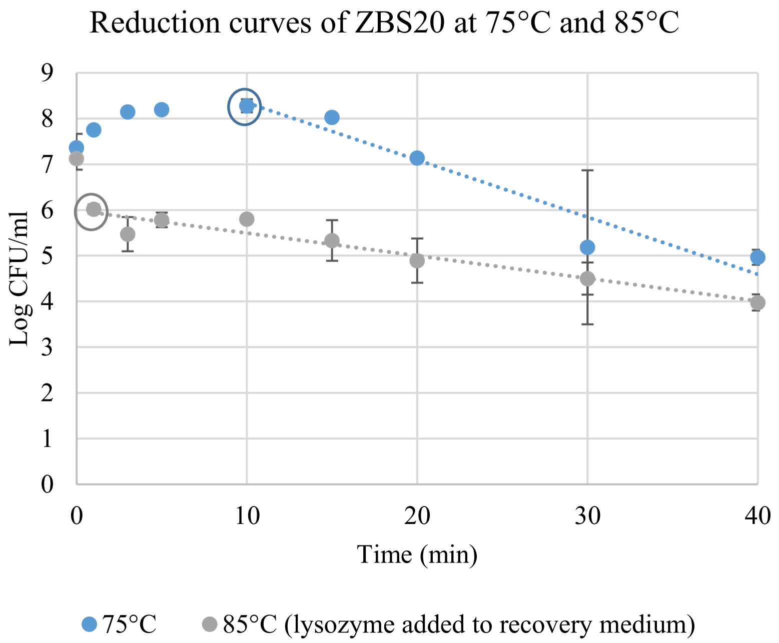

Reduction curves of ZBS20 at 75°C and 85°C
9
8
7
6
5
4
3
2
1
0
Log CFU/ml
0
10
20
30
40
Time (min)
75°C
85°C (lysozyme added to recovery medium)

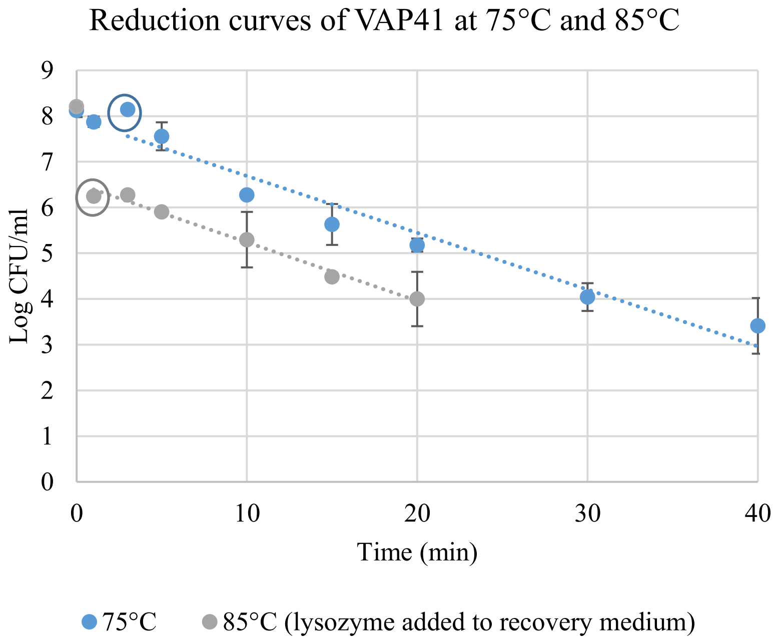

Reduction curves of VAP41 at 75°C and 85°C
9
8
7
6
5
4
3
2
1
0
Log CFU/ml
0
10
20
30
40
Time (min)
75°C
85°C (lysozyme added to recovery medium)

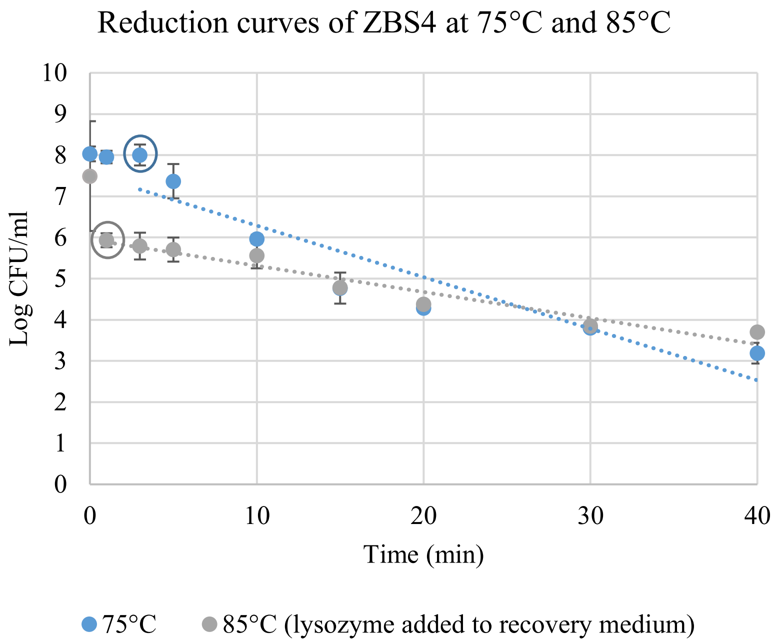
Reduction curves of ZBS4 at 75°C and 85°C
Log CFU/ml
Time (min)
0
1
2
3
4
5
6
7
8
9
10
0
10
20
30
40
75°C
85°C (lysozyme added to recovery medium)

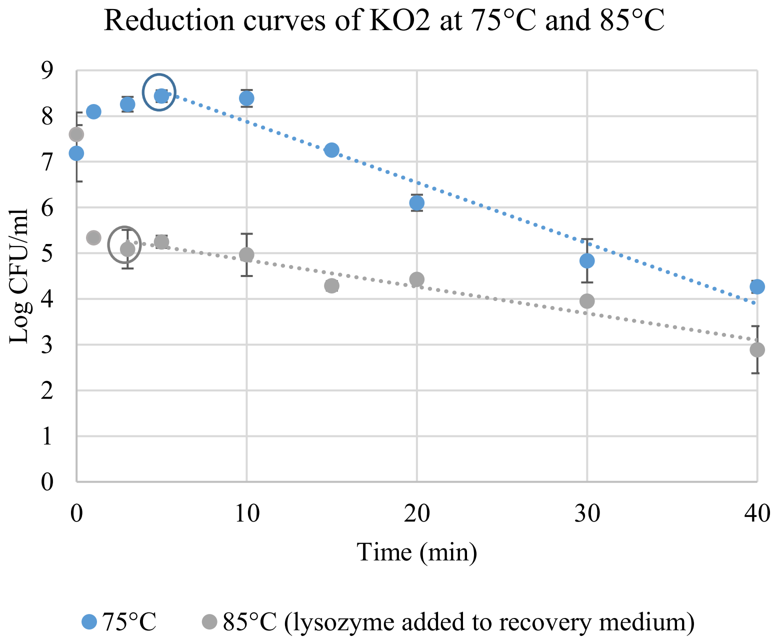
Reduction curves of KO2 at 75°C and 85°C
Log CFU/ml
Time (min)
0
1
2
3
4
5
6
7
8
9
0
10
20
30
40
75°C
85°C (lysozyme added to recovery medium)

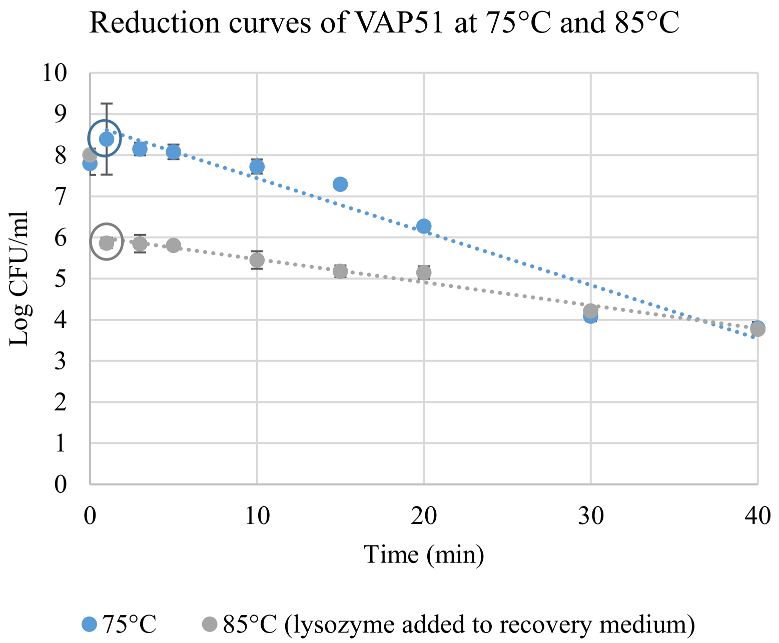
Reduction curves of VAP51 at 75°C and 85°C
Log CFU/ml
Time (min)
0
1
2
3
4
5
6
7
8
9
10
0
10
20
30
40
75°C
85°C (lysozyme added to recovery medium)

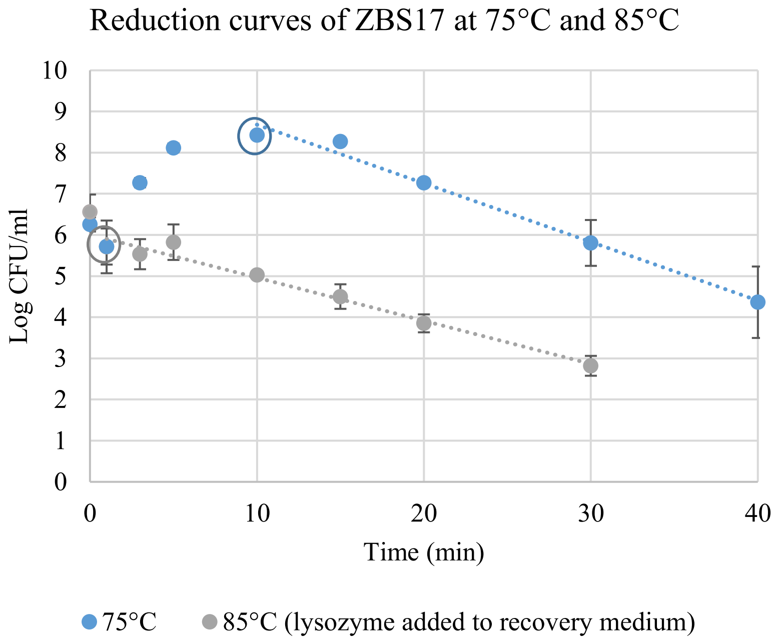
Reduction curves of ZBS17 at 75°C and 85°C
Log CFU/ml
Time (min)
0
1
2
3
4
5
6
7
8
9
10
0
10
20
30
40
75°C
85°C (lysozyme added to recovery medium)

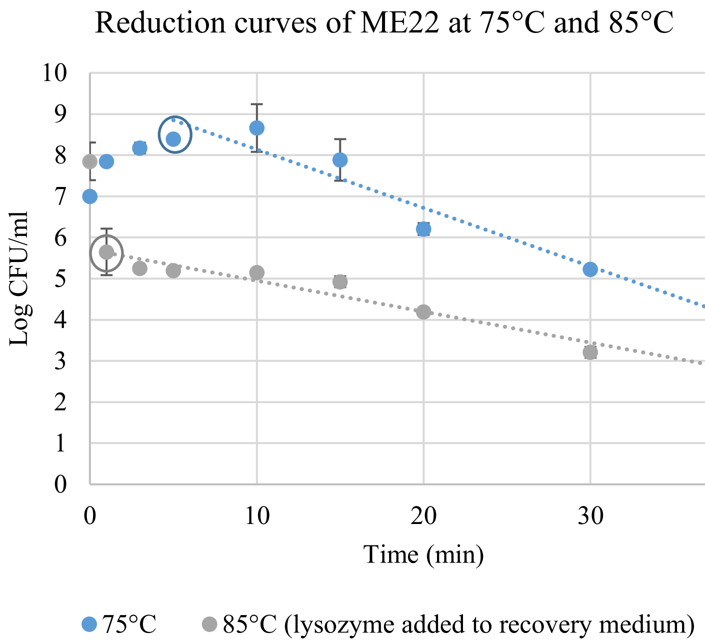
Reduction curves of ME22 at 75°C and 85°C
Log CFU/ml
Time (min)
0
10
20
30
40
0
1
2
3
4
5
6
7
8
9
10
75°C
85°C (lysozyme added to recovery medium)

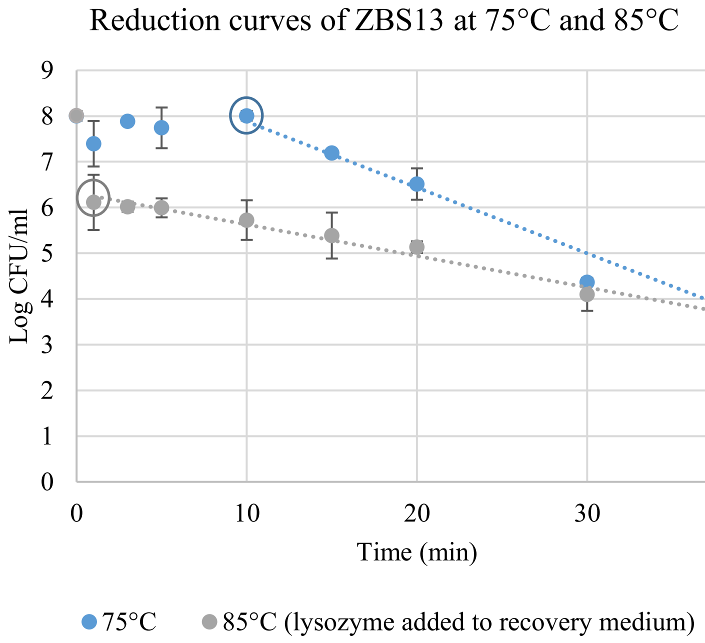
Reduction curves of ZBS13 at 75°C and 85°C
Log CFU/ml
Time (min)
0
10
20
30
40
0
1
2
3
4
5
6
7
8
9
75°C
85°C (lysozyme added to recovery medium)

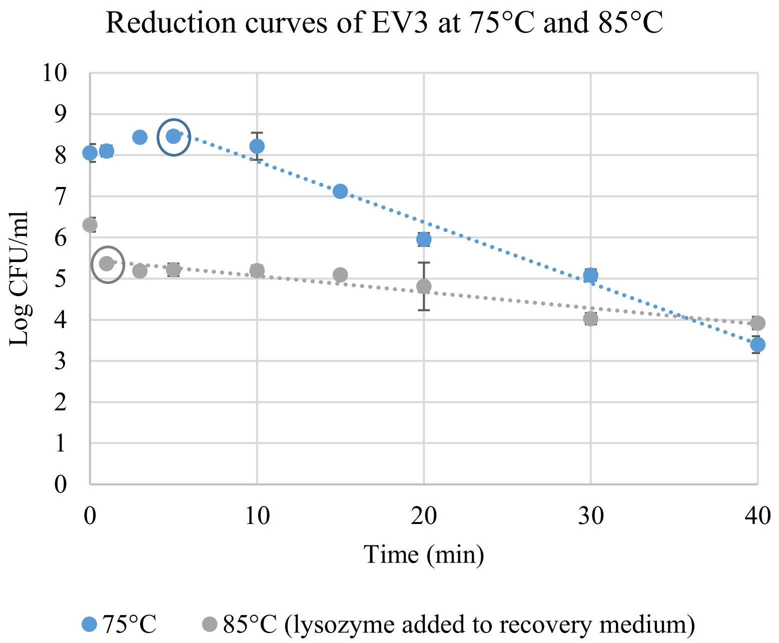
Reduction curves of EV3 at 75°C and 85°C
Log CFU/ml
10
9
8
7
6
5
4
3
2
1
0
0
10
20
30
40
Time (min)
75°C
85°C (lysozyme added to recovery medium)

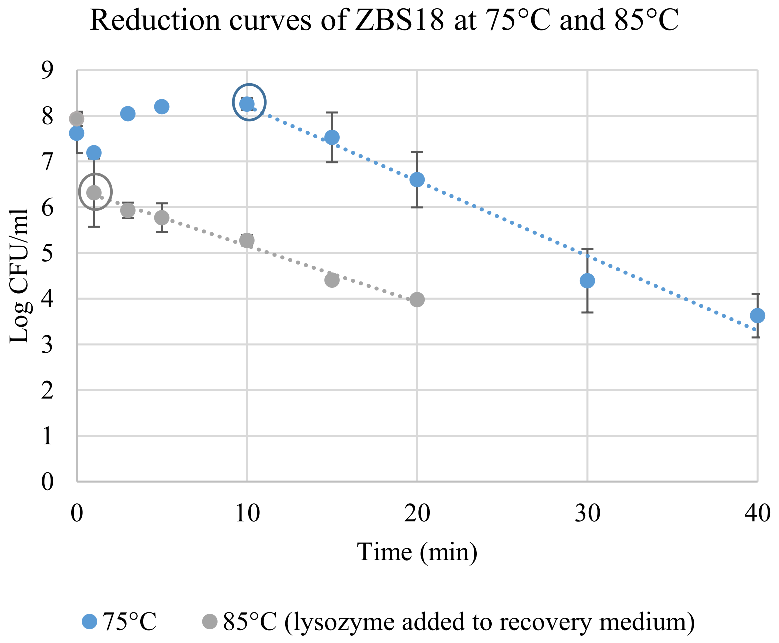
Reduction curves of ZBS18 at 75°C and 85°C
Log CFU/ml
9
8
7
6
5
4
3
2
1
0
0
10
20
30
40
Time (min)
75°C
85°C (lysozyme added to recovery medium)

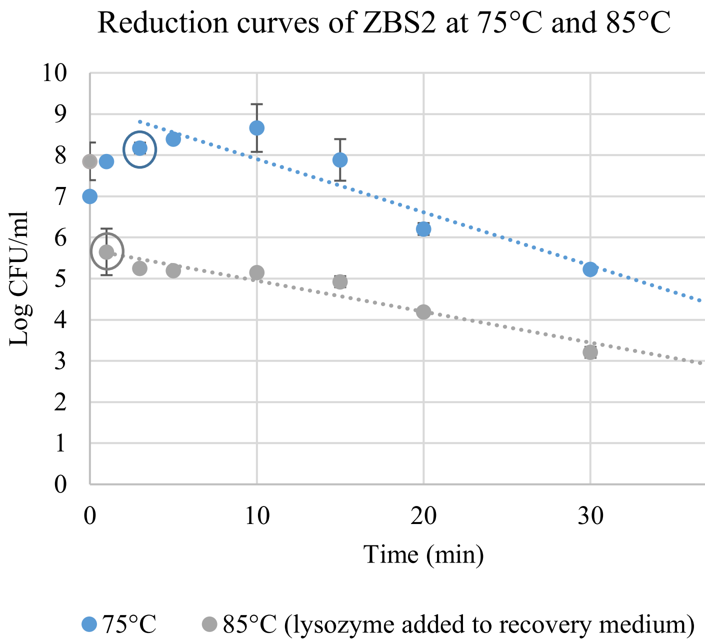
Reduction curves of ZBS2 at 75°C and 85°C
Log CFU/ml
Time (min)
0
1
2
3
4
5
6
7
8
9
10
0
10
20
30
40
75°C
85°C (lysozyme added to recovery medium)

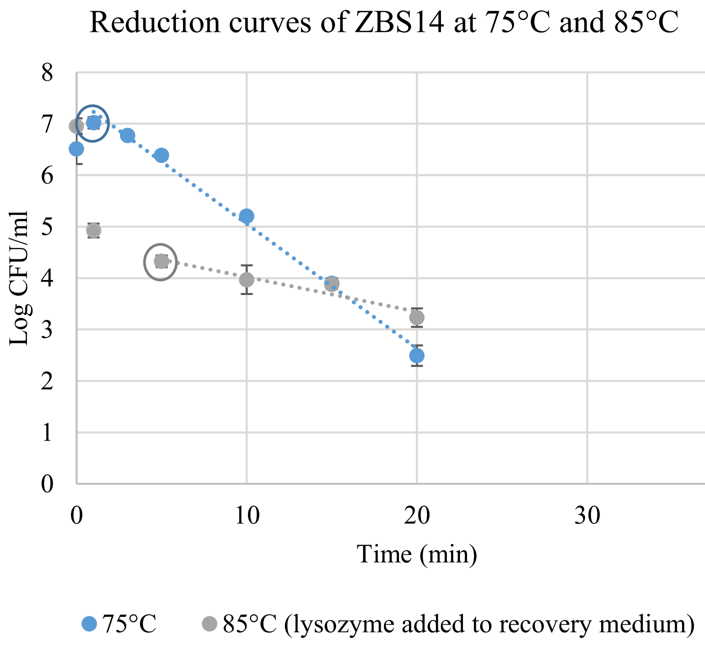
Reduction curves of ZBS14 at 75°C and 85°C
Log CFU/ml
Time (min)
0
1
2
3
4
5
6
7
8
0
10
20
30
40
75°C
85°C (lysozyme added to recovery medium)

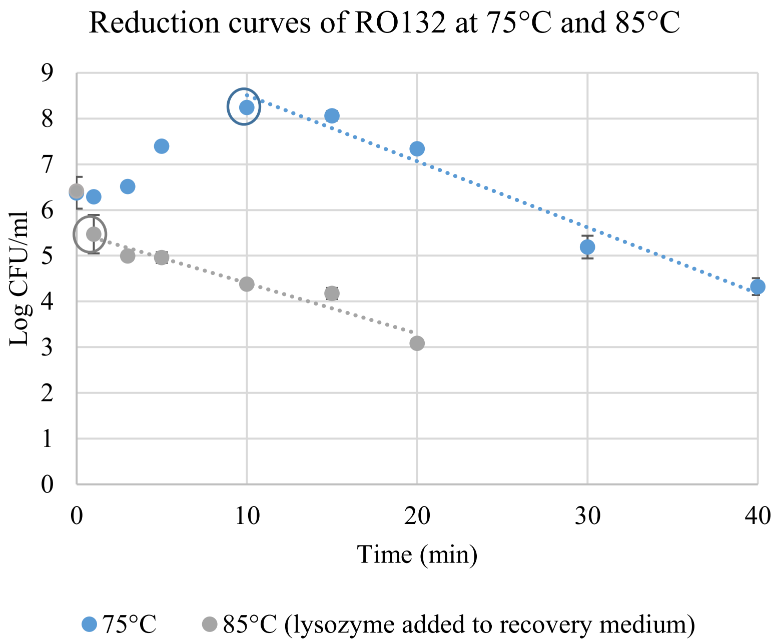
Reduction curves of RO132 at 75°C and 85°C
Log CFU/ml
Time (min)
0
10
20
30
40
0
1
2
3
4
5
6
7
8
9
75°C
85°C (lysozyme added to recovery medium)

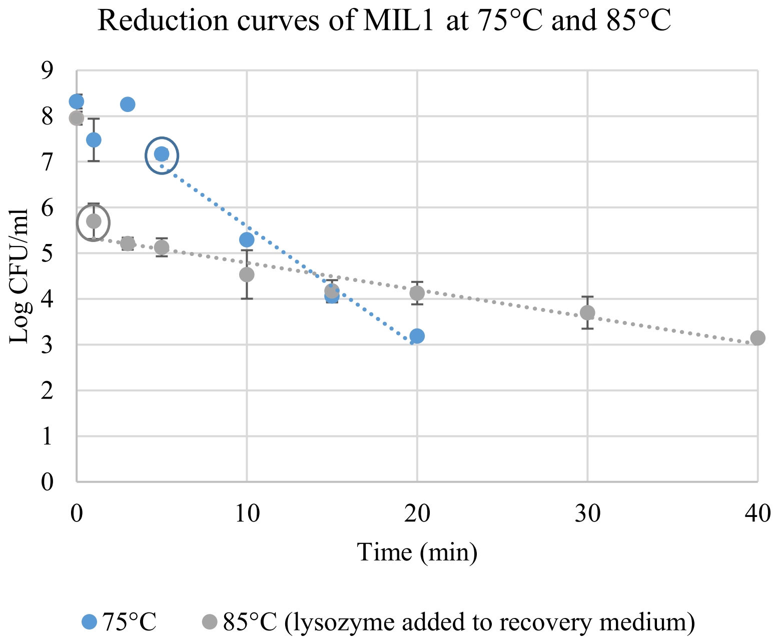
Reduction curves of MIL1 at 75°C and 85°C
Log CFU/ml
Time (min)
0
10
20
30
40
0
1
2
3
4
5
6
7
8
9
75°C
85°C (lysozyme added to recovery medium)

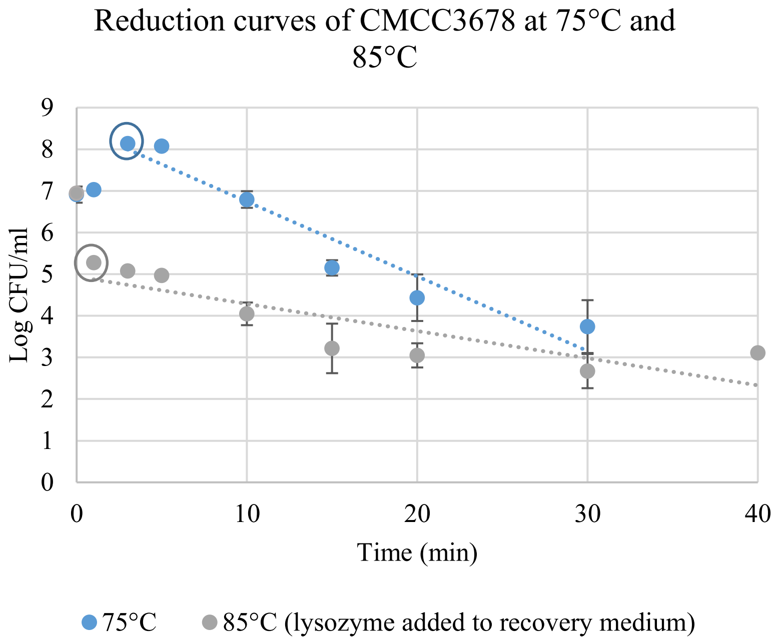
Reduction curves of CMCC3678 at 75°C and 85°C
Log CFU/ml
Time (min)
0
1
2
3
4
5
6
7
8
9
0
10
20
30
40
75°C
85°C (lysozyme added to recovery medium)

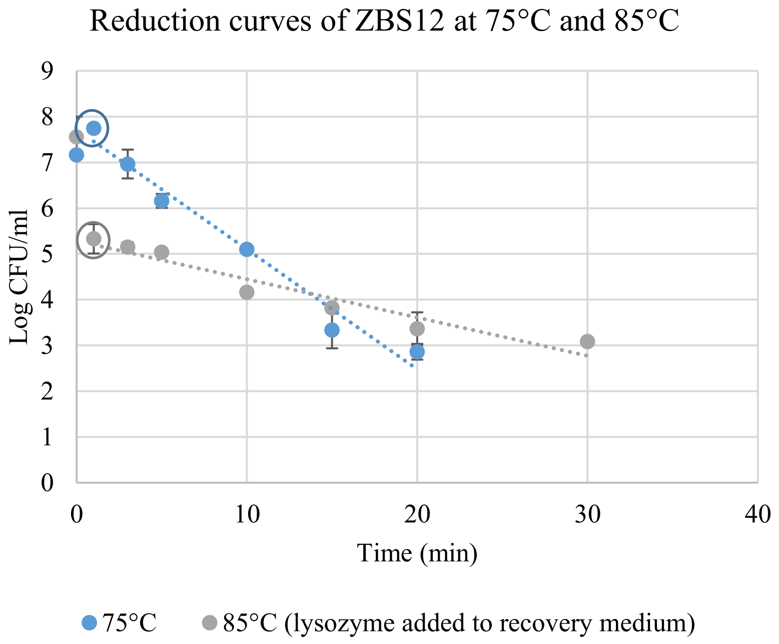
Reduction curves of ZBS12 at 75°C and 85°C
Log CFU/ml
Time (min)
0
1
2
3
4
5
6
7
8
9
0
10
20
30
40
75°C
85°C (lysozyme added to recovery medium)

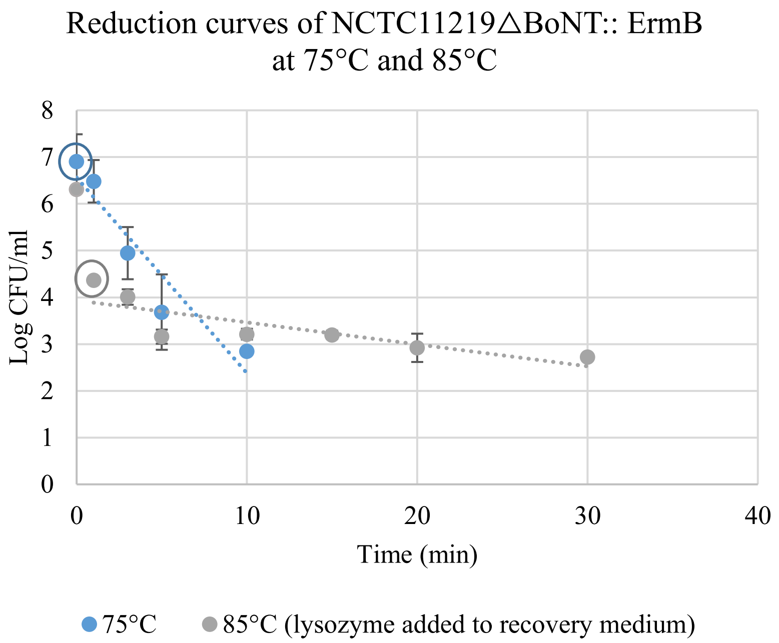
Reduction curves of NCTC11219△BoNT:: ErmB at 75°C and 85°C
Log CFU/ml
Time (min)
0
1
2
3
4
5
6
7
8
0
10
20
30
40
75°C
85°C (lysozyme added to recovery medium)

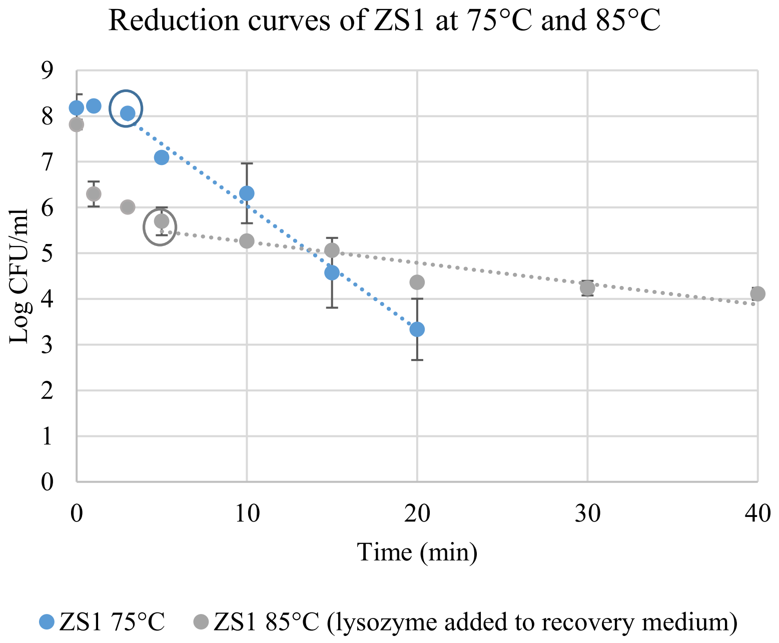
Reduction curves of ZS1 at 75°C and 85°C
Log CFU/ml
Time (min)
0
1
2
3
4
5
6
7
8
9
0
10
20
30
40
ZS1 75°C
ZS1 85°C (lysozyme added to recovery medium)

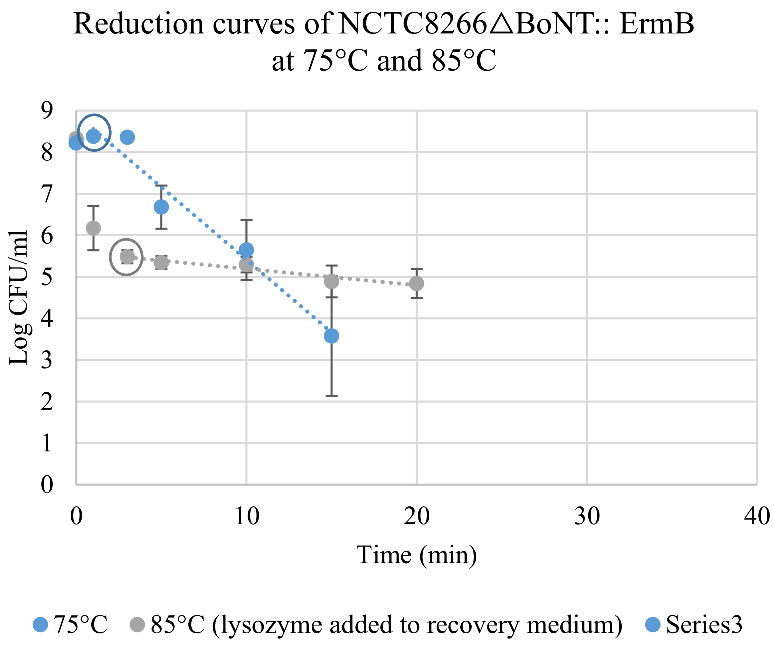

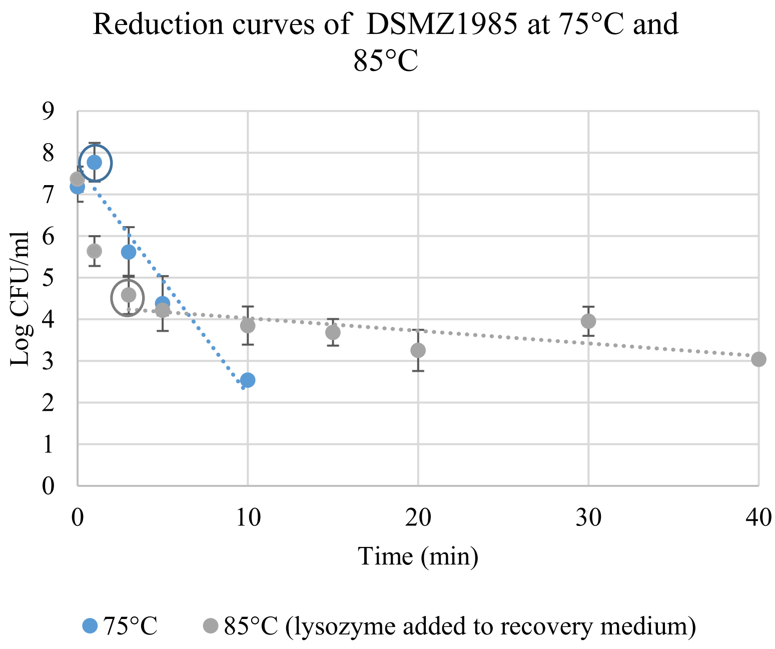

Figure S4 Reduction curves for heat inactivation of all nontoxic isolates, NCTC8266Δbont::ermB and NCTC11219Δbont::ermB. The recovery medium for spores treated at 85°C was supplemented with lysozyme (10 μg/ml). D-values of biphasic curves were calculated from the second part of the curve, taking the circled data point as the start. Average values ± STDEV from three replicates are shown.
